# Cryptic isoprene emission of soybeans

**DOI:** 10.1073/pnas.2502360122

**Published:** 2025-06-12

**Authors:** Mohammad Golam Mostofa, Abira Sahu, Yuan Xu, Insiya Basrai, Lior Doron, Violet Lefrancois, Thomas D. Sharkey

**Affiliations:** ^a^Department of Energy Plant Research Laboratory, Michigan State University, East Lansing, MI 48824; ^b^Department of Biochemistry and Molecular Biology, Michigan State University, East Lansing, MI 48824; ^c^Plant Resilience Institute, Michigan State University, East Lansing, MI 48824; ^d^Department of Chemistry, State University of New York College of Environmental Science and Forestry, Syracuse, NY 13235; ^e^Department of Environmental Health Sciences, University of Alabama, Birmingham, AL 35205

**Keywords:** isoprene, climate change, wounding, soybean

## Abstract

Isoprene is the most abundant nonmethane biogenic hydrocarbon emitted by some plants, mostly trees. It plays critical roles in atmospheric chemistry by contributing to ozone and aerosol formation. Isoprene also benefits plants, particularly under stress, through its signaling roles. Legume crops like soybean were thought to have evolutionarily lost isoprene synthase (ISPS) and are typically considered nonemitters. Here, we report that damage to soybean leaves by wounding or burning triggered a burst of isoprene emission from the undamaged part of the leaves. In silico analysis identified intact *ISPS* genes in the soybean genome, with features similar to known ISPSs. Protein made from these gene sequences catalyzed isoprene production in the presence of dimethylallyl diphosphate. Isoprene emission in soybeans was linked to reduced photosynthesis rates and stomatal conductance. Metabolomic analysis showed that leaf damage caused a surge in glyceraldehyde 3-phosphate and pyruvate levels, leading to an increase of most of the methylerythritol 4-phosphate pathway metabolites.

## Significance

Isoprene emission from plants is thought to be the largest hydrocarbon input to the atmosphere. Crop plants generally do not emit isoprene, but we now show that soybeans do emit isoprene in response to leaf wounding and high temperatures. Leaf wounding also reduced photosynthetic capacity by reducing rubisco (the CO_2_ fixing enzyme) activity. We found many isoprene synthases (ISPS) within the bean family (Fabaceae) of plants and confirmed that two putative ISPS of soybeans make isoprene but at much lower rates than typical isoprene-emitter. The findings will help shape our thinking about isoprene’s functions in crop resilience and growth–defense tradeoffs.

Plants emit a diverse set of volatile organic compounds with isoprene being one of the most abundant. Isoprene emission from plants constitutes a major flux of hydrocarbons (1) contributing up to 660 tera-grams of reactive carbon to the atmosphere annually (2, 3), surpassing inputs from anthropogenic sources. This makes isoprene a substantial player in global atmospheric chemistry. Isoprene is linked to the production of tropospheric ozone and aerosols, and can extend the half-life of methane, collectively triggering air pollution that impacts environmental and human health (3–5). However, under low abundance of nitrogen oxides (NOx), isoprene can suppress the formation of ozone and aerosols (4, 6). Despite its significance in the atmospheric environment, the biology and evolutionary origins of isoprene production in plants remain poorly understood. Thus, it is crucial to unravel the genetic and biochemical basis underlying isoprene emission from different plant species to better comprehend the evolutionary advantages and ecological roles of isoprene.

Isoprene synthesis is mostly found in hard-wood trees (e.g., poplars and eucalyptus), mosses (e.g., *Campylopus introflexus* and *Sphagnum* spp), and some legumes (e.g., *Pueraria montana* and *Macuna pruriens*) (7–11). It was previously thought that modern crops, including soybean (*Glycine max*), do not emit isoprene. Measurements of soybean leaf gas exchange did not find isoprene and soybeans were thought to lack an intact *isoprene synthase* (*ISPS*) gene (3). However, a recent genome construction (12) revealed two intact *ISPSs* in soybeans, raising a question of why they do not emit isoprene. Could soybean ISPS be active only under specific environmental stresses, as seen in native soybean (*Glycine soja*) (3)? Or might soybeans emit isoprene during extreme conditions like heat waves to confer cellular benefits?

Isoprene emission is linked to enhanced plant resilience under various stresses, including heat (13–19), salinity (20), oxidative stress (21–23), and herbivory (24–26). Emitting plants maintain higher photosynthetic efficiency and growth rates under heat and osmotic stresses (14, 17, 23, 27, 28). Interactions of isoprene with phytohormones like jasmonic acid (JA) (29, 30) and cytokinin (CK) (20, 31, 32) suggest its regulatory roles in stress responses. Additionally, isoprene-responsive genes contain cis-regulatory elements associated with stress signaling pathways (33) and phosphoproteomics reveals its impact on proteins involved in photosynthesis, metabolism, and membrane dynamics (34). These beneficial effects weigh against the energy and carbon cost of isoprene production such that the net effect is positive for some species, in which case *ISPS* is retained, but net negative in other species, and these species lost their *ISPS* or never acquired one.

Soybean is a crucial crop in global agriculture, serving diverse purposes from animal feed to industrial applications. Given the global focus on sustainable agriculture and food security, the importance of soybeans continues to be paramount. Therefore, understanding the dynamics of soybean production, including environmental concerns, is crucial for stakeholders in agriculture, trade, and environmental policy. Soybeans have been traditionally considered nonemitters of isoprene (7) and the potential impact of soybean isoprene emission to the atmosphere has not been integrated into global isoprene models (2, 3). It is likely that climate associated factors like heat waves and rapid insect infestations may further increase isoprene flux from crop fields to the environment. Indeed, soybean fields are facing the intensifying impact of these climatic factors (35, 36) signifying the importance of careful evaluation of soybean physiology in relation to isoprene emission.

Here, we identified an unlikely phenomenon in soybeans, termed “cryptic isoprene emission,” triggered under specific environmental pressures. We carried out genome-wide, in-silico analysis across legume and nonlegume species to confirm the presence of *ISPS* gene(s) in the soybean genome. Molecular cloning and subsequent characterization of soybean *ISPS*, *terpene synthase* (*TPS)8 (TPS8)* and *TPS23*, revealed their functional roles in isoprene emission. Mechanical damage-induced isoprene emission was correlated with physiological responses in soybean leaves, while metabolite profiling linked the methylerythritol 4-phosphate (MEP) pathway and photosynthesis-related metabolites to this isoprene burst. Our findings lay the groundwork for understanding the underlying mechanism of wound-induced isoprene emission in soybeans, and perhaps in other legume crops harboring *ISPSs*. This study also provides clues for studying crop-mediated isoprene emission effects on the atmosphere and whether crop plants can harness the benefit of isoprene’s signaling roles in adaptation to climate change.

## Results

### Evolutionary Origin of Cryptic ISPS in Soybeans.

**Fig. 1.**
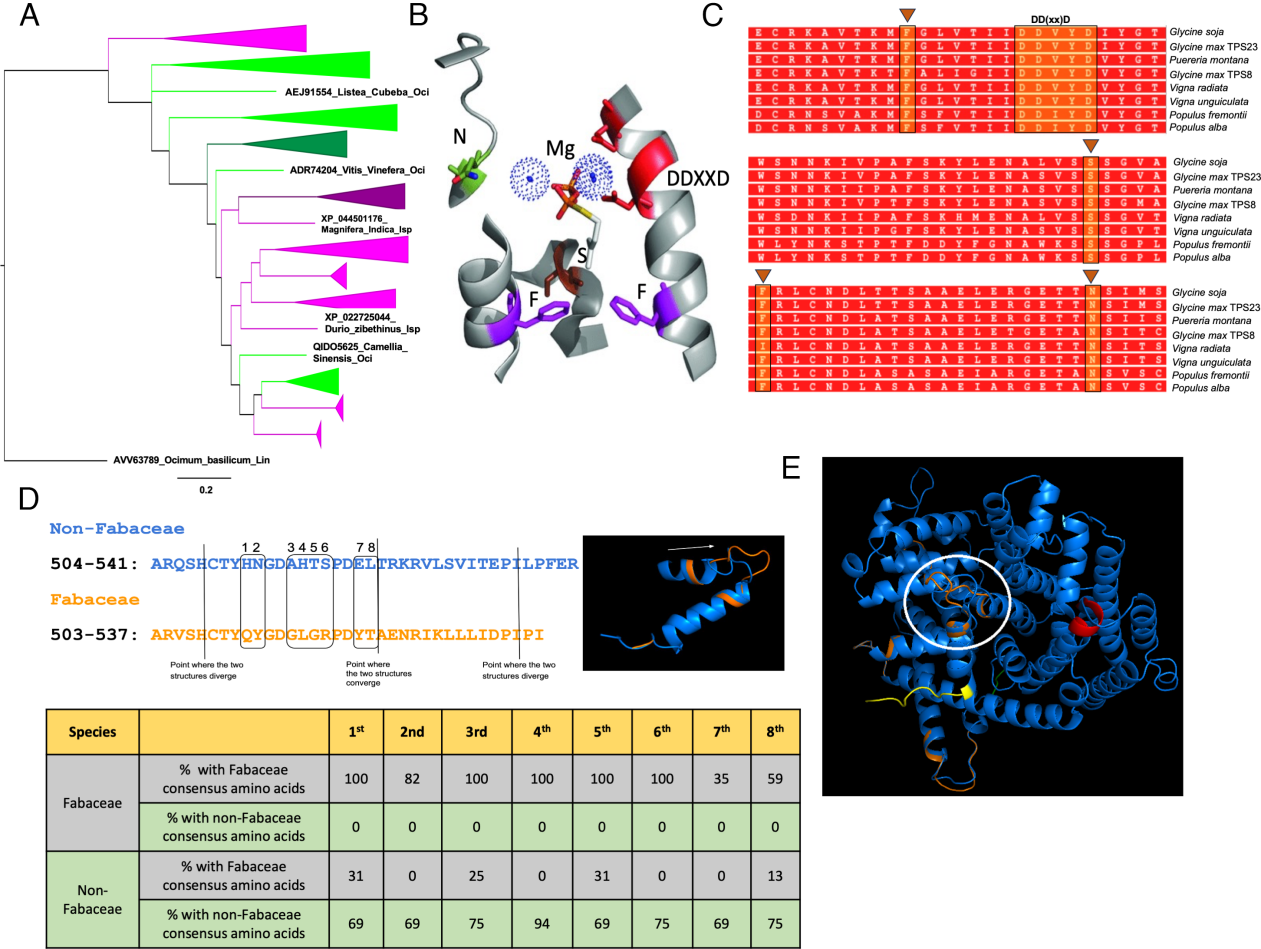
In-silico analysis of ISPS in Fabaceae and non-Fabaceae family. (*A*) Phylogenetic tree of ISPS gene sequences (purple) and *E-β-ocimene synthase* gene sequences (green). The darker shades are used for the Fabaceae sequences. The sequences were aligned in CLC Genomics Workbench, and the tree was estimated in MrBayes with 3 million generations using 82 total sequences. The final SD value was 0.00943. The resulting tree was drawn in FigTree (version 1.4.4). (*B*) Structural features of ISPS (PDB 3NOG). Four amino acids, including two phenylalanine (F), serine (S), and asparagine (N) are specific to ISPSs, whereas the DDXXD motif and 2 magnesium (Mg) atoms are conserved in all terpene synthases (TPSs). (*C*) Sequence alignment of *Populus* ISPS with Fabaceae ISPSs reveal the presence of all characteristic amino acids (F, S, F, and N) and DDXXD motif in all legumes including soybean ISPSs (TPS23 and TPS8). (*D*) Alignment of consensus ISPS sequences of Fabaceae and non-Fabaceae indicates the structural difference between the two families. (*E*) Superimposed 3D structures of consensus ISPS proteins of Fabaceae (orange ribbon) and non-Fabaceae (blue ribbon) family reveals a distinct loop in the Fabaceae ISPS.

BLAST search showed a sporadic distribution of ISPSs and ocimene synthases across Fabaceae and non- Fabaceae families (Fig. 1*A* and *SI Appendix*, Fig. S1). Species that have both ISPS and ocimene synthases include *G. soja, Quercus robur, Quercus lobata, Spatholobus suberectus, Cajanus cajan, Vigna unguiculata, Arachis ipaensis, Arachis stenosperma, Arachis duranensis,* and *Abrus precatorius*. However, *P. montana,* known to be a prolific isoprene emitter, did not have residues that might explain the differences in kinetics.

Genera with species that possess ocimene synthases, but not ISPS include *Vitis, Juglans, Carya, Camellia, Phaseolus,* and *Lotus* (*SI Appendix*, Fig. S1). Species that possess ISPS but no ocimene synthase include *Glycine max, P. montana var. lobata, Mucuna pruriens, Sphenostylis stenocarpa, Vigna radiata var. radiata, Wisteria sp., Robinia pseudoacacia, Arachis hypogaea, Mangifera indica,* and all known sequences from *Populus* (Fig. 1*A* and *SI Appendix*, Fig. S1). Fabaceae ISPS split into their own clade relatively early (Fig. 1*A*), separating Fabaceae from non-Fabaceae ISPS and all ocimene synthases.

### Structural Variation of Soybean Cryptic ISPS from ISPSs of Natural Emitters.

Alignment of GmTPS8 and GmTPS23 with known ISPSs from *Populus* revealed that key amino acid residues (F, S, F, and N) and the metal binding motif DDXXD, typically present in all ISPSs (7), are conserved in GmTPS8 and GmTPS23 (Fig. 1 *B* and *C* and *SI Appendix*, Fig. S2). These features are also present in other legumes, including *G. soja*, *P. montana*, *V. radiata*, and *V. unguiculata*. (Fig. 1*C* and *SI Appendix*, Fig. S2). Additionally, Fabaceae ISPSs displayed a substitution of eight amino acid residues (QYGLGRYT) compared to the non-Fabaceae family (Fig. 1*D*). A distinct loop in between 504 to 541 amino acid residues was observed in Fabaceae ISPS, indicating a structural difference between Fabaceae and non-Fabaceae ISPS (Fig. 1*E*). However, *P. montana,* a Fabaceae species known to be a prolific isoprene emitter, did not have residues that might explain the differences in kinetics.

### Functional Validation of Soybean ISPS in Isoprene Synthesis.

**Fig. 2.**
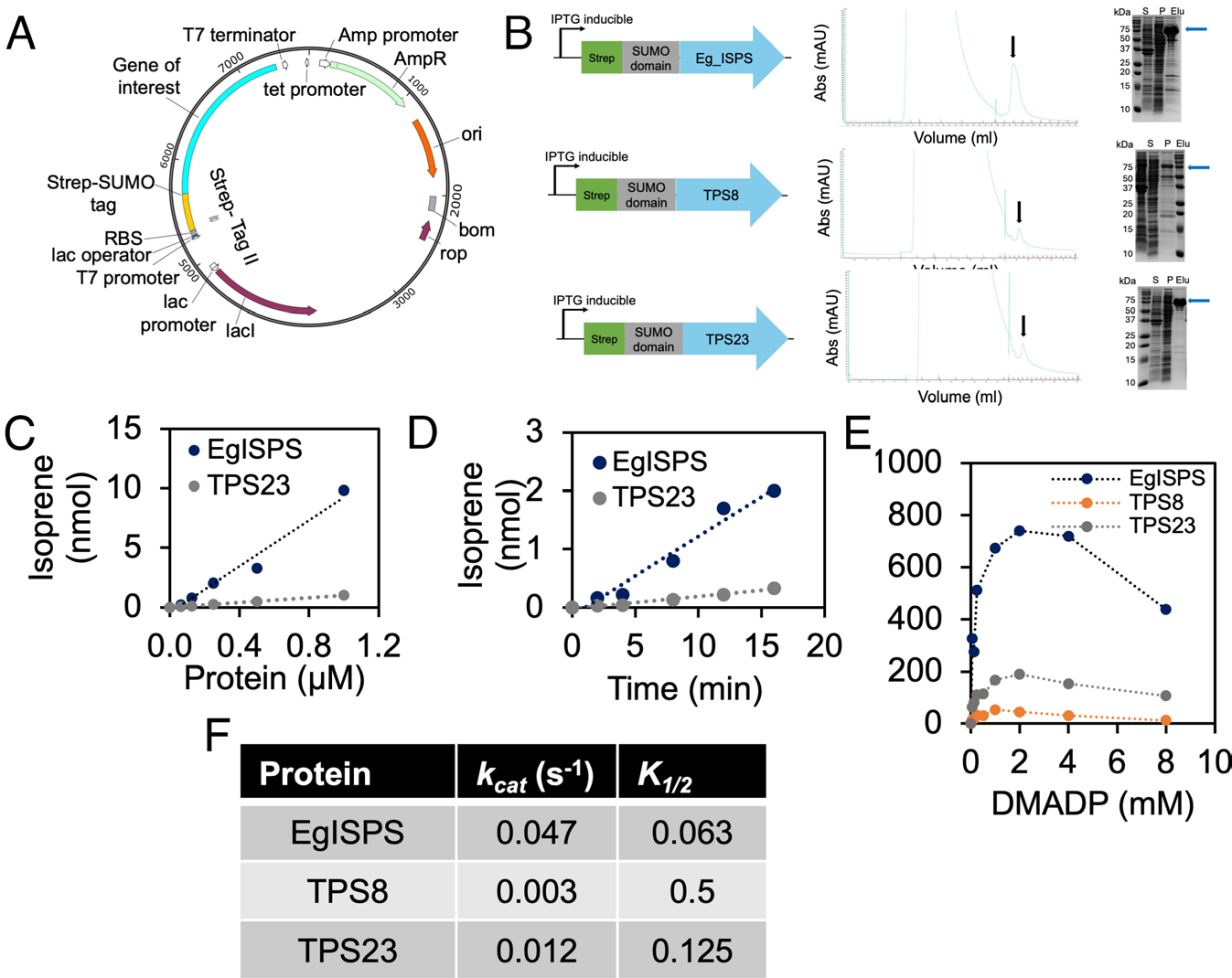
Expression and purification of recombinant soybean ISPS and in-vitro characterization of purified ISPSs. (*A*) ISPS genes from eucalyptus (*Eg_ISPS*) and soybean (*TPS8* and *TPS23*) were cloned to an IPTG-inducible expression plasmid followed by expression in *E. coli* BL21, and purification using a Strep affinity column. (*B*) Diagram of Strep-tagged ISPS genes and chromatogram of ISPS detected by Fast protein liquid chromatography (FPLC). Strep affinity tag (green rectangle) and SUMO domain (gray rectangle) were fused to the N-terminus of *ISPS* genes (blue arrow) downstream to an IPTG-inducible promoter. Elution peaks are marked by black arrows. SDS-PAGE analysis shows a specific band for each purified ISPS. (*C* and *D*) Isoprene emission capacity of EgISPS and TPS23 increases in a concentration and time-dependent manner. (*E*) Purified EgISPS, TPS8, and TPS23 show isoprene emission capacity in presence of different concentrations of dimethylallyl diphosphate (DMADP). (*F*) *k_cat_* and *K_1/2_*of purified EgISPS, TPS8, and TPS23 based on their catalytic performance in conversion of DMADP to isoprene. S: total soluble proteins before Strep affinity purification, P: insoluble fraction, Elu: pooled and concentrated elution fractions. ISPS peaks are marked with blue arrows.

To evaluate the catalytic function of soybean ISPSs*, GmTPS8*, *GmTPS23*, and *EgISPS* (control) were cloned and expressed in *Escherichia coli* BL21 using Strep-tag under IPTG-inducible promoter (Fig. 2 *A* and *B*). The overexpressed proteins of each ISPS were purified from the BL21 cells and eluted using Strep affinity column. Fast protein liquid chromatography (FPLC) and SDS-PAGE analyses showed clear peaks and bands for each of EgISPS, GmTPS23, and GmTPS8 (Fig. 2*B*). To test their capacity to emit isoprene and enzyme kinetics, EgISPS, GmTPS23, and GmTPS8 were incubated with the substrate dimethylallyl diphosphate (DMADP). GmTPS23 and EgISPS exhibited isoprene emission in a time- and DMADP-concentration (up to 2 mM) manner (Fig. 2 *C* and *D*). Both GmTPS23 and GmTPS8 showed increasing isoprene emission with rising DMADP concentrations up to 2 mM DMADP after which their activities declined due to substrate inhibition (Fig. 2*E*). GmTPS8 and GmTPS23 had 16-fold and 4-fold lower maximum turnover rates (*k_cat_*), respectively, much lower than EgISPS (Fig. 2*G*). Because of substrate inhibition, a *K_m_* could not be determined; instead, *K_1/2_‚* values were calculated to assess substrate affinity. Both GmTPS8 and GmTPS23 displayed significantly higher *K_1/2‚_* values suggesting lower binding affinity for isoprene than EgISPS (Fig. 2*F*). None of the enzymes exhibited cooperativity with increasing substrate concentrations.

### Cryptic Isoprene Emission in Soybean Leaves.

**Fig. 3.**
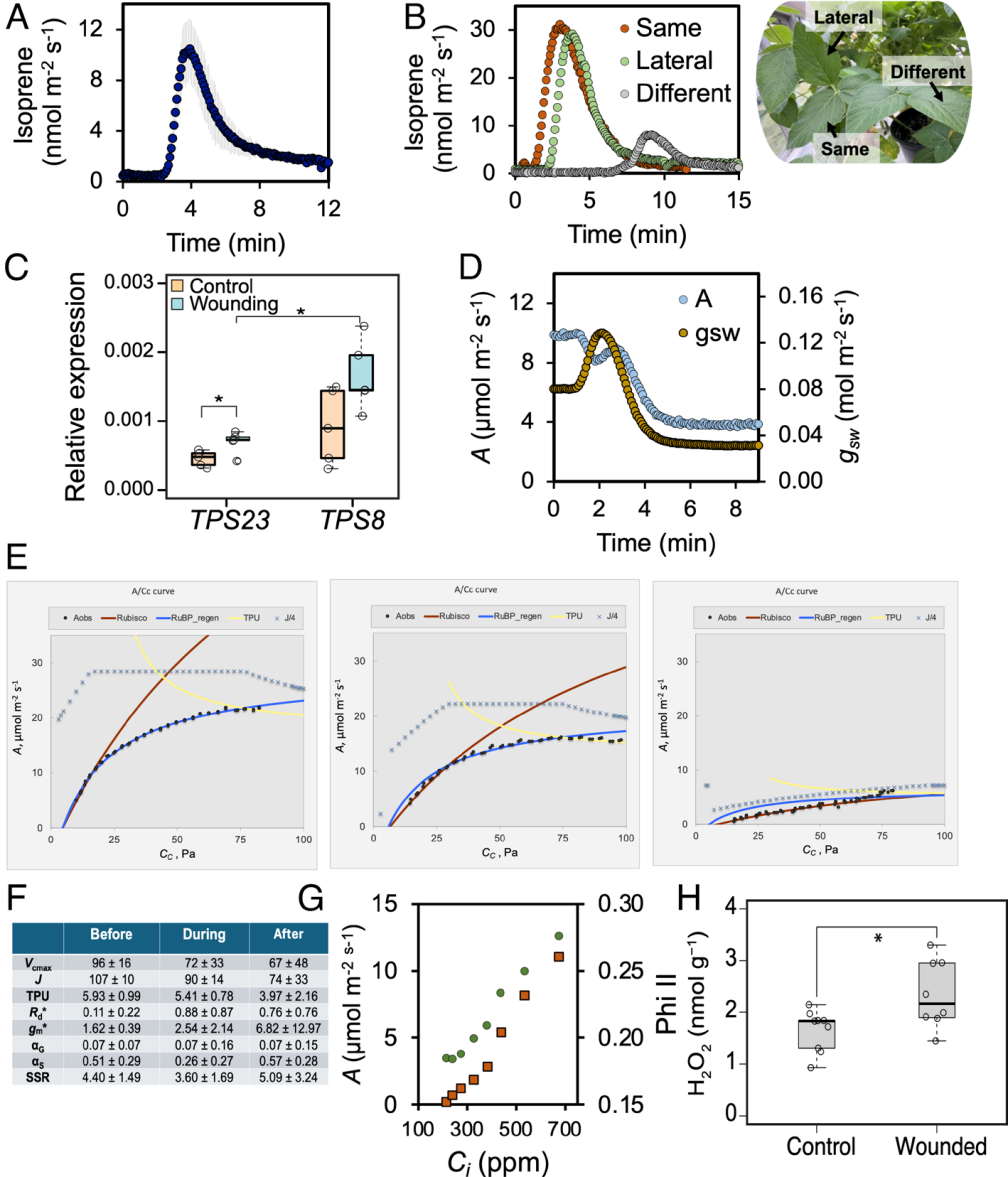
Cryptic isoprene emission and physiological, biochemical, and gene expression changes in soybean leaves. (*A*) Soybean leaves transiently emit isoprene following wounding. (*B*) Isoprene burst is also evident in soybean plants after burning of the same, lateral, and distant leaflets. (*C*) Expression levels of *GmTPS8* and *GmTPS23* genes following wounding of soybean leaves. (*D*) Isoprene emission accompanies differential responses of CO_2_ assimilation (*A*) and stomatal conductance (*g_sw_*) upon wounding. (*E*) *A/C_c_* response curves illustrate rubisco activity, ribulose-1,5-bisphosphate (RuBP) regeneration, and triose phosphate utilization (TPU) limitation in soybean leaves before, during, and after wounding. (*F*) Comparative responses of *A* in soybean leaves following wounding shows that photosynthesis was rubisco limited, even at the lowest *A* (*V_cmax_*= maximum rubisco activity, *R_d,_*= respiration during photosynthesis, RuBP regen = ribulose bisphosphate carboxylase regeneration, TPU = triose phosphate use limitation, *J* = rate of electron transport, *g_m_* = mesophyll conductance, α_G_ = amount of carbon that leaves the photorespiratory cycle as glycine, α_S_ amount of carbon that leaves the photorespiratory cycle as serine, SSR = sum of the squared residuals). (*G*) The level of hydrogen peroxide (H_2_O_2_) determined before and after wounding of soybean leaves (*n = 8*). (*H*) photosynthetic efficiency of photosystem II (Phi II). Asterisks indicate significant differences at *P* <0.05 according to Student’s *t* test.

Isoprene emission from soybean leaves was below the limit of detection under normal conditions but showed transient emission when damaged by wounding or burning (Fig. 3 *A* and *B* and *SI Appendix*, Fig. S5). Wounding induced a rapid increase in isoprene emission from the undamaged leaf parts, peaking at ~12 nmol m^−2^ s^−1^ within 4 min, followed by a gradual decline, stabilizing at ~2 nmol m^−2^ s^−1^ after 8 min (Fig. 3*A*). Burning triggered a stronger response, reaching ~30 nmol m^−2^ s^−1^ emission by 5 min before returning to baseline by 10 min (Fig. 3*B*). Burning a lateral leaflet induced similar levels of isoprene emission from an undamaged leaflet of the same trifoliate leaf while burning a distant leaflet on a different branch resulted in lower (~8 nmol m^−2^ s^−1^) and delayed response, peaking around 10 min (Fig. 3*B*). These results suggest that a wound-induced signal propagates throughout the plant and affects isoprene emission based on proximity to the wounding site. Isoprene production from wounded soybean leaves was also confirmed by GC–MS analysis (*SI Appendix*, Fig. S6). Gene expression analysis revealed that wounding upregulated *GmTPS23* and *GmTPS8*. Although *GmTPS8* maintained higher expression than *GmTPS23*, the latter exhibited stronger induction, aligning with its greater activity in catalyzing isoprene formation (Figs. 1*E* and 3*C*).

### Soybean Cryptic Isoprene Emission Was Accompanied by a Decline of Photosynthesis.

Upon wounding, photosynthetic parameters such as CO_2_ assimilation (*A*) and stomatal conductance (*g_sw_*) showed an initial desynchronized response, followed by a synchronized decline (Fig. 3*D*). CO_2_ assimilation dropped sharply around 2 min postwounding, while *g_sw_* exhibited a sharp increase at the same time. After these early peaks, both parameters steadily decreased, stabilizing at ~8 µmol m^−2^ s^−1^ for *A* and ~0.04 mol m^−2^ s^−1^ for *g_sw_* by 6 to 8 min (Fig. 3*D*). The pronounced decline in *g_sw_* indicates that stomatal closure may play a key role in the sustained reduction of photosynthetic rates following wounding.

To understand the mechanisms behind wound-induced photosynthesis decline, we evaluated the limitation of ribulose 1,5-bisphosphate (RuBP) regeneration by electron transport chain (*J*), rubisco activity limitation (*V_cmax_*), and TPU limitation using the dynamic assimilation technique of the LI-COR 6800 so that entire CO_2_ response curves could be made faster than the changes in isoprene emission (Fig. 3 and *SI Appendix*, Table S3). The observed data clearly fitted on the *J*, or RuBP regeneration limitation line (blue) when isoprene peaked followed by reduction along the *V_cmax_* (red) line post-burning and -wounding (Fig. 3 *E* and *F* and *SI Appendix*, Figs. S7 and S8). Slight TPU limitation was observed at the emission peak and both behaviors were reproducible, but the most obvious effect was a large decline in *V_cmax_* post damage, indicating rubisco deactivation (Fig. 3*E* and *F* and *SI Appendix*, Table S3). To further confirm whether there was any effect of RuBP regeneration limitation on photosynthesis postwounding, we estimated Phi II responses upon wounding at variable CO_2_ levels. An increase of Phi II with increasing CO_2_ (Fig. 3*G*) indicated that RuBP limitation and TPU were not the cause of photosynthetic reduction upon wounding. We measured H_2_O_2_ levels before and after wounding of soybean leaves. Significant accumulation of H_2_O_2_ was detected postwounding (Fig. 3*H*), which may be a mechanism for altering the growth (rubisco activity) - defense (stimulation of ISPS) tradeoff.

### Wounding and Burning Resulted in Activation of Metabolic Flow Through the MEP Pathway.

**Fig. 4.**
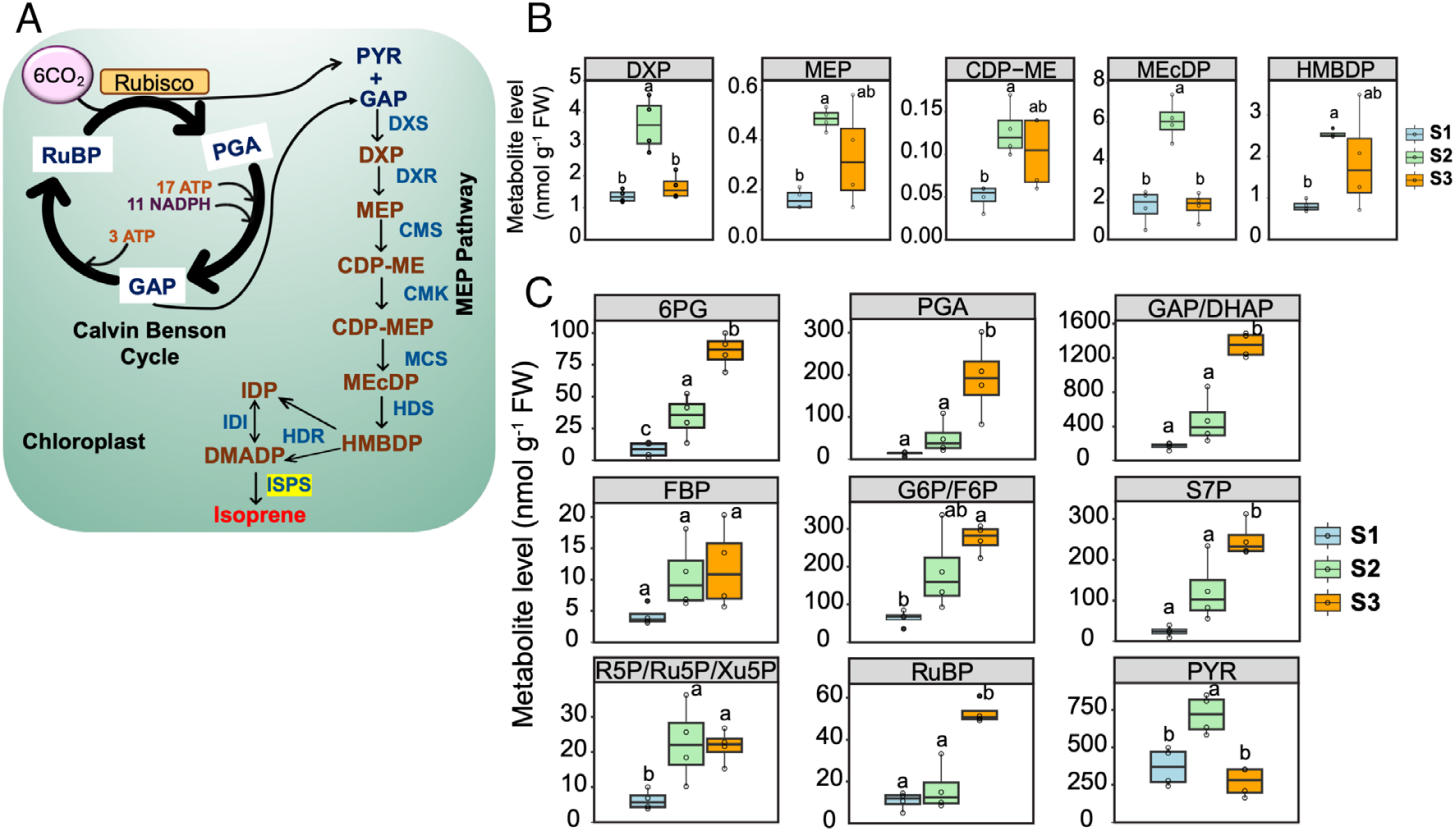
Wound-induced changes in MEP pathway and photosynthesis related metabolites. (*A*) Isoprene biosynthesis precursors originated from Calvin Benson cycle (CBC). Using pyruvate (PYR) and glyceraldehyde 3-phosphate (GAP), dimethylallyl diphosphate (DMADP) is synthesized from MEP pathway products by enzyme-mediated reactions and ultimately converted to isoprene by ISPS. (*B*) The levels of MEP pathway metabolites, including DXP, MEP, CDP-ME, MEcDP, and HMBDP were determined in soybean leaf samples collected before (S1), during (S2), and after (S3) wounding. (*C*) Calvin-Benson Cycle provides the precursors of PYR and GAP. The levels of metabolites, including 6-phosphogluconate (6PG), GAP/dihydroxyacetone phosphate (DHAP), fructose 6-phosphate (FBP)/glucose 6-phosphate (G6P), glucose 6-phosphate/fructose 6-phosphate (G6P/F6P), sedoheptulose 7-phosphate (S7P), ribose 5-phosphate (R5P)/ ribulose 5-phosphate (Ru5P)/xylulose 5-phosphate (Xu5P), ribulose 1,5-bisphosphate (RuBP), and PYR were measured in soybean leaf samples before (S1), during (S2), and after (S3) wounding. DXP, 1-deoxyxylulose 5-phosphate; MEP, 2*-C-*methyl-d-erythritol 4-phosphate; CDP-ME, 4-diphosphocytidyl-2*-C-*methyl-d-erythritol; CDP-MEP, 4-diphosphocytidyl-2*-C-*methyl-D-erythritol 2-phosphate; MEcDP, 2*-C-*methyl-D-erythritol 2,4-cyclodiphosphate; HMBDP, 1-hydroxy-2-methyl-2-(*E*)-butenyl 4-diphosphate; DMADP, dimethylallyl diphosphate; IDP, isoprenyl diphosphate; DXS, 1-deoxyxylulose-5-phosphate synthase; DXR 1-deoxyxylulose-5-phosphate reductoisomerase; CMS, 4-diphosphocytidyl-2*-C-*methyl-d-erythritol synthase; CMK, 4-diphosphocytidyl-2-*C*-methyl-D-erythritol 2-phosphate kinase; MCS, 2-*C*-methyl-D-erythritol 2,4-cyclodiphosphate synthase; HDS, 1-hydroxy-2-methyl-2-(*E*)-butenyl 4-diphosphate synthase, HDR, 1-hydroxy-2-methyl-2-(*E*)-butenyl 4-diphosphate reductase; IDI, isopentenyl diphosphate isomerase.

To understand the mechanism of increased isoprene emission after mechanical damage we measured the MEP pathway metabolites (Fig. 4*A*). Both wounding and burning caused a significant upregulation of most MEP metabolites (Fig. 4*A* and *SI Appendix*, Fig. S9). The levels of deoxyxylulose 5-phosphate (DXP), methylerythritol 4-phosphate (MEP), 4-(cytidine-diphospho)-2-C-methyl-D-erythritol (CDP-ME), methyl-D-erythritol 2,4-cyclodiphosphate (MEcDP), and 4-hydroxy-3-methylbut-2-enyl diphosphate (HMBDP) were significantly upregulated during the peak of isoprene emission upon wounding (S2) compared to prewounding (S1). At postwounding (S3), DXP and MEcDP returned to prewounding (S1) level while MEP, CDP-ME, and HMBDP remained elevated. Likewise, significant enhancement of DXP and MEP levels was detected postburning, while CDP-ME, MEcDP, and HMBDP showed a similar pattern although not statistically significant (*SI Appendix*, Fig. S9).

To check the status of MEP pathway precursors pyruvate and glyceraldehyde 3-phosphate (GAP) (Fig. 4*A*), we examined the levels of several metabolites involved in central carbon metabolism which exhibited dynamic changes upon wounding and burning (Fig. 4*C* and *SI Appendix*, Fig. S9). The levels of 6 phosphogluconate (6-PG), pyruvate, and ribose 5-phosphate/ribulose 5-phosphate/xylulose 5-phosphate (R5P/Ru5P/Xu5P) significantly increased upon wounding (S2). While pyruvate returned to base level, 6-PG and R5P/Ru5P/Xu5P remained significantly higher at S3. The other metabolites, including 3-phosphoglycerate (PGA), GAP/dihydroxyacetone phosphate (DHAP), fructose 1,6-bisphosphate (FBP), glucose 6-phosphate/fructose 6-phosphate (G6P/F6P), sedoheptulose 7-phosphate (S7P), and ribulose 1,5-bisphosphate (RuBP) also increased at S2 and continued increasing significantly (except FBP) at S3. Similar change in these metabolite levels was observed upon leaf burning (*SI Appendix*, Fig. S9). In particular, 6PG, PGA, GAP/DHAP, FBP, G6P/F6P, S7P, R5P/Ru5P/Xu5P, and pyruvate levels significantly increased at S2 relative to S1 followed by returning to their base levels at S3 (*SI Appendix*, Fig. S9). Overall, wounding and burning induced an upregulation of MEP pathway precursors, particularly pyruvate, indicating that central carbon metabolism undergoes a significant shift toward MEP pathway metabolism.

### Wounding-Induced Isoprene Emission Showed Connection with Elevated JA.

To reveal whether wound-induced isoprene has any correlation with the levels of phytohormones, we determined the levels of JA and JA-related metabolites 12-oxo-phytodienoic acid (OPDA), JA-isoleucine (JA-Ile), and methyl JA (MeJA), salicylic acid (SA), SA-conjugate SAG, and abscisic acid (ABA) (*SI Appendix*, Fig. S10). OPDA levels were significantly higher in S2 and S3 compared with S1. JA, JA-Ile, and MeJA exhibited a gradual increase, showing significantly higher levels at S3 relative to S1. ABA levels remained relatively stable across all stages, with no significant differences observed (*SI Appendix*, Fig. S10). In contrast, SA shows a marked increase in S3, with S1 and S2 maintaining significantly lower and comparable levels. SAG levels remained consistent across S1, S2, and S3. These results indicate that phytohormones associated with stress and defense, particularly OPDA, JA, and JA-Ile, are upregulated in the postwounding stage (S3), reflecting a robust response to wounding. Meanwhile, ABA and SAG appear to remain unaffected by the wounding process (*SI Appendix*, Fig. S10).

### Cryptic Isoprene Emission Under Extreme Environments.

We examined the levels of wound-induced isoprene emission from soybean leaves under elevated heat and CO_2_. Although wounding triggered isoprene emission in soybean leaves under both ambient and high CO_2_ conditions, emission levels showed a significant decrease under elevated CO_2_ and heat stress with greatest decrease observed under their combined conditions (Fig. 4*A*). We estimated that isoprene synthesis in wounded leaves used ~2% of assimilated carbon while under high climatic conditions it used ~1% (Fig. 4*B*).

We tested the response of cryptic isoprene emission under short-term and long-term heat stress. In the short term, increasing the temperature from 30 °C to 38 °C in the LI-COR chamber resulted in a marked increase of isoprene emission from soybean leaves (Fig. 4 *C* and *D*). Emission declined gradually when the temperature was reverted to 30 °C, approaching initial levels. For long-term heat stress, we measured isoprene emission from plants kept in a 42 °C growth chamber (Fig. 4 *E*–*G*). After 2 h, isoprene emission was significantly higher at 42 °C compared to 25 °C and 30 °C (Fig. 4*F*). The response intensified further after 20 h heat exposure (Fig. 4*G*). These results demonstrate that prolonged exposure to high temperature enhances isoprene emission from soybean leaves over time.

## Discussion

The evolutionary capacity for isoprene emission has long been debated (37), highlighting its roles in plant adaptation to heat flecks and oxidative stress. This study reveals that mechanical wounding and burning induce isoprene emission in soybeans. Our findings are particularly surprising given the previous assumption that soybeans lack functional isoprene emission capacity.

Earlier genomic studies (Williams 82, v1) posited that soybeans lost their capacity for isoprene emission, leaving behind apparent pseudogenes for ISPS (7). Our in-silico analysis using a reconstructed soybean genome (Williams 82, v4) revealed two intact ISPS genes, *TPS8* and *TPS23* (Fig. 1 *A*–*C*), contradicting the previous paradigm. Comparative phylogenetic analyses of ISPSs and ocimene synthases in Fabaceae and non-Fabaceae families offer deeper insights into their evolutionary relationships. Ocimene synthases form a monophyletic clade (Fig. 1*A* and *SI Appendix*, Fig. S1), indicating a shared evolutionary origin distinct from ISPSs. This suggests that ocimene synthases may have evolved from ancestral ISPSs, given that ISPS represents a basal, ancestral condition within this evolutionary framework (Fig. 1*A* and *SI Appendix*, Fig. S1). Intriguingly, many sequences previously labeled as “tricyclene synthases EBOS [(E)-*β*-ocimene synthases] were found to share homology with ocimene synthases rather than tricyclene synthases (7). Previous phylogenetic analyses indicated that ISPSs and ocimene synthases share homology, but ISPSs and tricyclene synthases do not (7, 38). We believe that the EBOS stands for (E)-*β*-ocimene synthases and that all these sequences were mislabeled and are actually ocimene synthases. Moreover, our results suggest two relatively recent evolutionary events of Fabacean ISPS re-emergence from Meso-Papilionoideae and Phaseoleae clades (*SI Appendix*, Fig. S1).

The function of ISPSs in angiosperms is strongly associated with the presence of two key Phe residues, homologous to F338 and F485 in *P. alba*. These residues are critical for shaping the enzyme’s active site and ensuring obligate isoprene production (7). F338 closes the rear of the active site, while F485 secures the H-helix side, preventing alternative binding with GDP (39–41). Interestingly, in the *Humulus lupulus* myrcene synthase, which lacks the F485 equivalent, F354 (homologous to F338) alone supports isoprene production, albeit less efficiently, due to suboptimal active site configuration (7). This suggests that the presence of both Phe residues enhances, but may not be strictly required for, isoprene synthesis. In soybeans, TPS8 and TPS23 contain both Phe residues alongside other residues like serine and asparagine (N) (S445 and N505 in *P. alba*) and the conserved DDXXD motif (Fig. 1 *B* and *C* and *SI Appendix*, Fig. S2). We synthesized both putative ISPSs in soybean and found they both made isoprene from DMADP (*SI Appendix*, Fig. S3). These results suggest that soybean ISPSs have the required conformation to catalyze isoprene production. Nevertheless, a specific motif “GLGR,” which is close to the active site, is present in Fabaceae but absent in non-Fabaceae ISPSs (Fig. 1 *D* and *E*), possibly marking a signature of cryptic ISPS. These motifs and residues could be diagnostic tools for studying ISPS functionality and evolutionary adaptation in Fabaceae.

This structural difference near the active site, might hinder soybeans’ TPS8 and TPS23 ability to catalyze isoprene synthesis from DMADP efficiently despite having all the characteristic features of ISPS. Indeed, purified GmTPS8 and GmTPS23 catalyzed DMADP to isoprene in vitro at much slower rate than EgISPS (Fig. 2*E*). Moreover, both GmTPS8 and GmTPS23 exhibited a drop in isoprene production beyond 2 mM DMADP (Fig. 2*E*), suggesting substrate inhibition, as also observed in *Eucalyptus* and *Populus* ISPSs (7, 42). However, in measurements up to 16 mM this phenomenon was not seen in *P. montana*, a close relative of *G. max* (39). The lower *k_cat_* and high *K_1/2_* values of GmTPS8 and GmTPS23 compared to EgISPS highlight their lower catalytic efficiency and lower affinity for DMADP (Fig. 2*F*), supporting a slower rate of isoprene production (Fig. 2 *C*–*E*). Because of the low affinity, it is possible to have almost no isoprene emission at levels of DMADP sufficient for other isoprenoid biosynthesis. *P. montana* ISPS showed a high *K_m_* and strong cooperativity (39), which was not observed in GmTPS8 and GmTPS23. Thus, the kinetics of GmTPS8 and GmTPS23 seems to differ from that of *P. montana*, which is a prolific emitter (39).

Soybeans emit a burst of isoprene, but only transiently, when the same or nearby leaves on the same plant were wounded or burned (Fig. 3 *A* and *B* and *SI Appendix*, Figs. S4 and S5). This response also coincided with wound-induced expression levels of *TPS8* and *TPS23* (Fig. 3*C*). Similarly, isoprene emission was induced in wild soybean *G. soja’*s leaf upon burning the leaflet for five seconds (3). Also, *G. soja* has an active ISPS with a very high sequence similarity to the coding parts of the *G. max ISPS* genes (7). Isoprene emission was also evident in other legumes, such as *P. montana* and *Mucuna pruriens* (3). Within these legumes, isoprene emission followed a differential pattern. While *P. montana* emits constitutively, *G. soja* and *G. max* emit isoprene only upon wounding or burning (39) (Fig. 3 *A* and *B*).

Meanwhile, the undamaged parts of the leaves experienced a decline in photosynthetic rate (*A*) and stomatal conductance (*g_sw_*) when isoprene was emitted in response to wounding or burning (Fig. 3*D*). *A/C_i_* response curves revealed that wounding and burning caused RuBP regeneration limitation (lower *J*) at the initial stage, but a clear deactivation of rubisco activity (lower *V_cmax_*) dominated post wounding and burning (Fig. 3 *E* and *F* and *SI Appendix*, Figs. S6 and S7). Since Phi II increased in parallel with photosynthetic rate under a series of CO_2_ levels, the cause of the decline in photosynthesis postdamage is unlikely to be RuBP regeneration/electron transport chain activity or the triose phosphate utilization (TPU) limitation (Fig. 3*G*). A signal caused by burning traveled from the damaged part of the leaf to the part of the leaf in the gas exchange cuvette. This signal can even travel from different leaflets and different leaves (Fig. 3*B*). A similar response was also observed in velvet bean (*Mucuna pruriens*) (43), but in that case the leaves were already emitting isoprene at the time of wounding. In soybeans, wounding resulted in a significant rise of H_2_O_2_ (Fig. 3*H*). H_2_O_2_ is a well-known inhibitor of rubisco activity directly (44) or indirectly by activating glucose 6-phosphate dehydrogenase (G6PDH) (45), which causes the accumulation of 6-PG (Fig. 4*C*), a potential inhibitor of rubisco when present in high concentration (46). Furthermore, an elevated level of RuBP (Fig. 4*C*) can also inhibit rubisco activity by binding to the inactive form of rubisco, preventing its association with CO_2_ and Mg^(2+)^ (46).

The MEP pathway is the source of the isoprene precursor DMADP (Fig. 4*A*). Our data indicated that a surge of CBC-metabolites, particularly GAP and pyruvate, activated the MEP pathway, as reflected in the levels of all measurable MEP pathway intermediates (Fig. 4 *B* and *C*). The increased CBC metabolites, including RuBP, was simultaneous with a reduction in photosynthetic activity (Figs. 3*D* and 4*C*). GmISPSs exhibit a low *K_1/2_* (high substrate requirement and low substrate affinity) and *k_cat_* (low catalytic efficiency) so that there is essentially no isoprene emission until DMADP builds up to the millimolar (mM) range. Competing enzymes for DMADP in the stroma (e.g., geranyl diphosphate synthase and adenylate isopentenyltransferase) have high substrate affinity, thus low micromolar (µM) DMADP level is enough to start the catalysis process (47, 48). Thus, the kinetics of GmISPSs ensure that isoprene emission does not compete with other reactions that require DMADP. These kinetics also reduce the cost of isoprene emission (20 ATP and 14 NADPH/isoprene) (15), since it is effectively turned off when not needed.

**Fig. 5.**
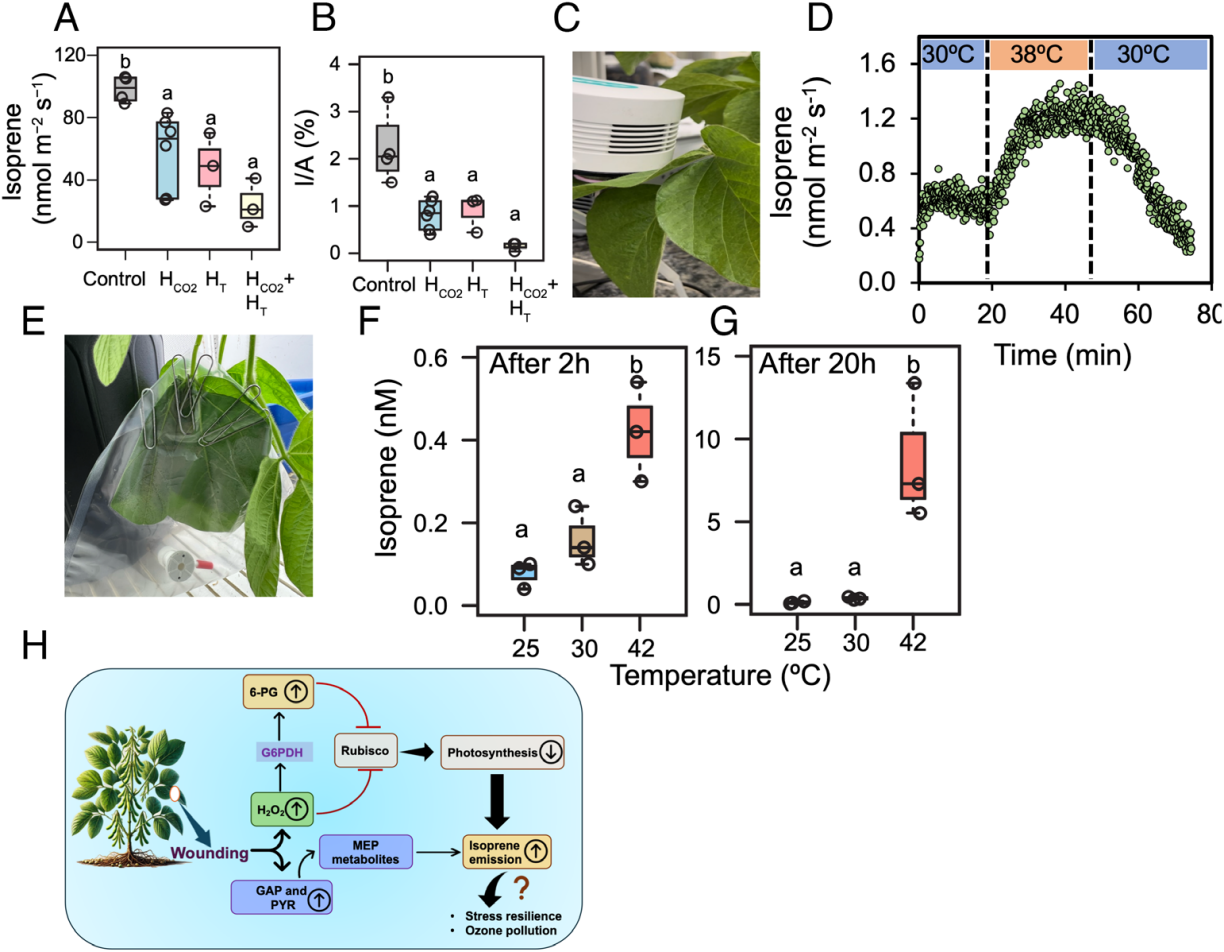
Isoprene emission in soybean leaves under different climate factors. (*A*) Effect of elevated CO_2_ (H_CO2_) and high temperature (H_T_) on wound-induced isoprene emission from soybean leaves. (*B*) Carbon utilization for isoprene synthesis at ambient vs. high CO_2_ and temperature conditions in wounded leaves. (C and *D*) Change in isoprene emission from soybean leaves during short-term heat stress (38 °C). (*E*–*G*) Isoprene emission from soybean leaves at 25, 30, and 42 °C after 2 h and 20 h incubation in growth chambers. (*H*) Mechanistic overview of cryptic isoprene emission and photosynthesis regulation upon wounding of soybean leaves. PYR, pyruvate; GAP, glyceraldehyde 3-phosphate; G6PDH, glucose-6-phosphate dehydrogenase; 6-PG, 6-phosphogluconate.

Climate factors like elevated CO_2_ and temperature interact dynamically with isoprene emission. Elevated CO_2_ suppresses isoprene synthesis by inhibiting MEP pathway enzyme HMBDP reductase (49), while high temperatures enhance emission (13). In constitutively emitting poplar, high temperature minimized the high CO_2_ isoprene suppression (49). Whereas in cryptic emitter soybean, an additive effect of elevated CO_2_ and high temperature resulted in further isoprene suppression (Fig. 5*A*). This intense effect further coincided with less carbon utilization for isoprene synthesis (Fig. 5*B*). Our findings suggest that wound-induced cryptic isoprene emission will be reduced under those elevated climate factors. We also noticed a significant isoprene emission when soybean plants were exposed to heat stress for both short-term (2 h) and long-term (20 h) (Fig. 5 *C*–*G*). Since isoprene plays a pivotal role in heat stress tolerance and has interactions with defense related hormones (*SI Appendix*, Fig. S9) in plants, can soybean “have its cake and eat it too?”, present when needed but turned off when not needed by altering the growth/defense tradeoff often seen in plant behavior. It is plausible that short-term heat stress triggers a rapid increase in isoprene emission to protect soybean leaves from transient damage. However, a prolonged heat stress sustains elevated isoprene emission, which may impose metabolic costs and ozone pollution to the atmosphere (Fig. 5*H*).

Taken together, our findings underscore the dynamic evolutionary trajectory of soybean ISPSs, with evidence supporting their ancient origin and subsequent diversification from ocimene synthases. The identification of cryptic isoprene emission in soybeans, coupled with evidence of photosynthesis regulation (Fig. 5*H*), provides a perspective on its ecological functions in the context of soybean resilience to climate factors and global isoprene emission.

## Methods

### Genome-Wide, In-Silico Analysis of ISPS Genes Across *Fabaceae* and Non-*Fabaceae* Families.

The Basic Local Alignment Search Tool (BLAST) was used to find ISPS proteins in both Fabaceae and nonfabacean families. From the search, a *G. max* ISPS (XP_014628074) was identified and a further BLAST with XP_014628074 resulted in a number of isoprene and ocimene synthases. There are similar sequences identified as “triterpene synthase EBOS”. We believe the EBOS stands for E-β-ocimene synthase and many of these entries had been labeled as such in the past. We treat the designation of “triterpene synthase EBOS” as E-β-ocimene synthases. A phylogenetic tree of isoprene and ocimene synthases was generated with amino acid sequences using MrBayes (version ver. 3.2.7a, Phylogeny.fr). Even though they are not labeled as such in the database, the sequences from *Eucalyptus globulus* (AB266390) and *Melaleuca alternifolia* (AY279379), which are well-known ISPSs (7), were included in the phylogenetic tree. A phenylalanine residue seven amino acids upstream from the DDXXD motif is diagnostic for ISPS (7), so any putative ISPS sequences that did not possess this characteristic were removed from the tree. A *S. stenocarpa* unnamed protein product (CAJ1977811) possessed this phenylalanine residue and was included. Ocimene synthases from *Rutaceae* (*Citrus*), Cannabaceae (*Cannabis*), *Brassicaceae* (*Arabidopsis*), *Calycanthaceae* (*Chimonanthus*), *Phrymaceae* (*Erythranthe*), and *Rosaceae* (*Prunus*) were added to contextualize the tree within previously estimated phylogenies.

The highly variable chloroplast transit sequence was excluded to increase accuracy, and each protein was represented only once for concision. A linalool synthase from *Vitis vinifera* was chosen as the outgroup since it is closely related to isoprene and ocimene synthases (7). The 82 sequences were aligned in the CLC Sequence Viewer, and inconsistencies (such as gaps or sequences that were too short) were edited out of the alignment. The sequences were analyzed in MrBayes (ver. 3.2.7a) with the Markov chain Monte Carlo method. Three million generations were run and the final SD of split frequencies was 0.00943. The resulting tree was drawn in FigTree (version 1.4.4) to color-code each family and add posterior probability values to each node.

### Evaluation of ISPS Structure.

The consensus structures of ISPS from Fabaceae and non-Fabaceae species were determined from consensus sequences. These sequences were obtained from respective alignments of ISPS amino acid sequences from Fabaceae and non-Fabaceae species in the CLC Genomics Workbench. The Fabaceae species used were *S. suberectus, C. cajan, A. precatorius*, *A. precatorius*, *A. ipaensis, A. duranensis, P. montana var. lobata [*kudzu*]*, *R. pseudoacacia, V. radiata var. Radiata, V. unguiculata, Glycine max, G. soja,* Wisteria *sp., A. stenosperma, A. hypogaea,* and *M. pruriens* (17 total). The non-Fabaceae species used were *Q. robur*, *Q. lobata, M. indica, M. indica, Eucalyptus grandis, Populus alba, Populus tremuloides, Populus grandidentata,* [*P. alba x Populus x berolinensis*], [*Populus tremula x P. alba*], *Populus deltoides*, *Populus fremontii*, *Populus balsamifera*, *Populus trichocarpa*, *Populus nigra*, and *Populus euphratica* (16 total). Any indeterminate amino acid residues (denoted by “X” in the consensus sequence) were replaced with an alanine (Ala) residue.

The sequences were rendered into protein structures using Phyre2 (ver 2.0), a protein fold recognition software whose data are supplied by the AlphaFold Protein Structure Database. The resulting structures were represented as ribbon models in PyMOL (ver. 4.6.0) and superimposed on one another. The segments of the amino acid sequence where the two models did not overlap were scrutinized to see whether there were any amino acid characteristics uniquely conserved by Fabaceae species or non-Fabaceae species. The percentage of species in each group that possess either the Fabaceae or non-Fabaceae consensus amino acid in a particular position was determined.

### Plant Materials, Growth Conditions, and Cultivation.

Healthy seeds of soybean (Williams 82) were sown in 20 L plastic pots (5 seeds/pot) containing Suremix soils (Michigan Grower Products, Galesburg, MI) under a greenhouse setting (16 h photoperiod, mean daily light integral 12 mol m^–2^ d^–1^, and day/night temperature 33 °C /22 °C). This greenhouse is located at 42°43′N, 84°28′W, East Lansing, MI. After gemination, three uniform seedlings were continuously grown in each pot. Plants were watered with half-strength Hoagland’s solution on every alternate day. Mature soybean leaves from the reproductive stage (R1, beginning of flowering) were used to carry out different set of experiments. Pots containing plants were brought from the greenhouse to the lab without going outside for conducting experiments and collecting leaf samples on the day of each experiment.

### Simultaneous Measurements of Gas Exchange Parameters and Isoprene Emission.

Gas exchange measurements were recorded using a LI-COR 6800 Portable Photosynthesis System (LI-COR Biosciences, Lincoln, NE). A fully expanded mature leaf was clamped into a 6 cm^2^ chamber and allowed to equilibrate under the conditions of 1,000 µmol m^–2^ s^–1^ photosynthetically active radiation (50% blue light and 50% red light), 30 °C, 420 µmol mol^–1^ CO_2_ (gases were mixed at different pressures and so are reported as mole fractions here), and 22 to 26 mmol mol^–1^ water vapor constrained by laboratory room temperature. Measurements of assimilation rate (*A*), stomatal conductance (*g_sw_*), and intercellular CO_2_ concentration (*C_i_*) were recorded for every 5 s. Exhaust air coming out of the LI-COR 6800 was directed to the Fast Isoprene Sensor (FIS; Hills Scientific, Boulder, Colorado) for simultaneous isoprene measurements (49) (*SI Appendix*, Fig. S4). The flow rate in the LI-6800 was set at 600 μmol s^–1^. Air exiting the LI-COR was directed to a Tee from which 70% (420 μmol s^–1^) of total flow was pulled into the FIS and the other arm with a short piece of tubing to provide a buffer so that there was always excess air beyond what the FIS was pulling. A 3.22 μmol mol^–1^ isoprene standard (Airgas, USA) was used for the FIS calibration. The background isoprene level was measured in the air flowing from the empty LI-COR chamber and was subtracted from the experimental readings. Isoprene measurements were averaged over 5 s intervals.

### Dynamic Assimilation Technique to Determine *A/C_i_* Response.

A fully expanded mature leaf was clamped in the 6 cm^2^ chamber of LI-COR 6800 and allowed to equilibrate under the conditions mentioned above. After photosynthesis reached steady state, wounding was inflicted on a part of the leaf outside the chamber and on the opposite side of the midrib using forceps. As isoprene emission began following wounding, the Dynamic Assimilation Technique (DAT) (50) was used to determine *A*/*Ci* response curve, as outlined by McClain et al. (51). This very rapid method allowed us to see the *A*/*Ci* behavior over short time periods. CO_2_ was first lowered to 50 µmol mol^–1^ and the leaf was acclimated to this CO_2_ level for 60 s. Then CO_2_ was ramped to 1,200 µmol mol^–1^ at the rate of 400 µmol mol^–1^ min^–1^. Atmospheric pressure was 98 kPa. The *A*/*Ci* data were analyzed using the method described by Sharkey (52).

### Leaf Tissue Collection for Metabolite Analysis.

Leaf tissue was collected using an apparatus called Fast Kill freeze clamp as described by Sahu et al. (49). A 6 × 6 cm chamber sealed with cling film wrap was attached to a modified LI-COR 6800 head and mounted on the Fast Kill apparatus. A uniform field of illumination was created by precisely positioning four gooseneck fiber optic illuminators. A hand-held digital light meter was used to check that light intensity of 1,000 µmol m^−2^ s^−1^ was maintained across the chamber. The leaf was tightly clamped in the chamber using bar clamps to prevent any leak and allowed to equilibrate under the conditions mentioned above. A thin wire thermocouple was used to monitor the leaf temperature. When both isoprene and assimilation rates stabilized, the thermocouple was removed and then two copper dies cooled in liquid nitrogen were driven through the Cling Wrap windows using a pneumatic cylinder. The time between interruption of the light and leaf temperature below 0 °C was 30 ms. We collected samples using the gas exchange chamber/freeze clamp at three time points (*SI Appendix*, Fig. S4); i) before burning/wounding when there was no isoprene emission, S1; ii) when isoprene emission reached its peak after burning/wounding, S2; and iii) when isoprene emission dropped to its baseline level post burning/wounding. Frozen leaf discs were immediately stored at –80 °C for analysis of metabolites, including various phytohormones.

### MEP Pathway Metabolite Analysis by Liquid Chromatography–Tandem Mass Spectrometry (LC–MS/MS).

#### Preparation of leaf extract.

Frozen leaf discs were ground into a fine powder using a tissue homogenizer. The cryoblock was chilled in –80 °C so that the leaf tissues remained frozen during the grinding process. Ice-cold extraction buffer, 300 μL, (3:1:1 acetonitrile: isopropanol: 20 mM ammonium bicarbonate adjusted to pH 10 with ammonium hydroxide) was added to the ground plant material and kept in ice for 15 min. They were then centrifuged at 4 °C at 14,000×*g* for 10 min. The supernatant, 200 µL, was aliquoted into glass inserts placed in 2 mL glass vials for LC–MS/MS analysis. The leaf extracts were analyzed by HPLC immediately after extraction.

#### Measurement of MEP pathway metabolites.

Measurements were conducted as described by Sahu et al. (49). Standards of the MEP pathway metabolites 1-deoxy-D-xylulose-5-phosphate (DXP), 2-C-methyl-D-erythritol-4-phosphate (MEP), 4-(cytidine 5′-diphospho)-2-C-methyl-D-erythritol (CDP-ME), 2-C-methyl-D-erythritol-2,4-cyclodiphosphate (MEcDP), and (E)-4-Hydroxy-3-methylbut-2-enyl diphosphate (HMBDP) were purchased from Echelon Biosciences (Logan, UT). An InfinityLab Poroshell 120 HILIC-Z, P column (2.1 × 100 mm, 2.7 micron) connected to a Xevo TQ-XS mass spectrometer was used to separate the compounds. For the mobile phase, we used 20 mM ammonium bicarbonate adjusted to pH 10.0 with ammonium hydroxide and acetonitrile. The column temperature was maintained at 25 °C. The mass-spectra acquisition setup included negative mode electrospray ionization mode, capillary 1.00 kV, source temperature of 150 °C, and desolvation temperature of 400 °C. A binary gradient was established using mobile phase A (20 mM NH_4_HCO_3_ in H_2_O, pH ~10) and mobile phase B (acetonitrile) at a flow rate of 0.2 mL/min. The gradient profile was as follows: 0.00 to 2.00 min, 20% A and 80% B; 2.00 to 6.00 min, 60% A and 40% B; 6.00 to 8.00 min, 60% A and 40% B; 8.10 to 10.00 min, 20% A and 80% B.

### CBC and Related Metabolite Analysis.

#### Preparation of leaf extract.

The extraction protocol was carried out following the method described by Xu et al. (53, 54) with slight adjustments. Frozen leaf discs were ground into a fine powder using a tissue homogenizer, then extracted with 3:7 chloroform: methanol for 2 h at 20 °C with mixing by vortexing every 30 min. Known concentrations of internal standard of D-[UL-^13^C_6_] fructose 1, 6-bisphosphate and nor-valine were added to the tube. To extract the water-soluble metabolites, 300 μL water was added to the tube before the extraction. The extract was vortexed for 20 s followed by centrifugation at 4,200×*g* for 10 min. The upper methanol-water phase was aliquoted into vials for LC–MS/MS. Polar extracts were divided into several aliquots from the upper layer, lyophilized to dryness, and stored at −80 °C before undergoing GC-MS or LC–MS/MS analyses.

#### Measurement of CBC metabolites by ion-pair chromatography – tandem mass spectrometry (IPC–MS/MS).

Measurements were conducted as described by Xu et al. (53). Freshly reconstituted lyophilized extract aliquots were introduced into an ACQUITY UPLC pump system (Waters, Milford, MA) coupled with Waters XEVO TQ-S UPLC/MS/MS (Waters, Milford, MA). Separation of the CBC metabolites were achieved by a 2.1 × 50 mm Acquity UPLC BEH C18 Column (Waters, Milford, MA) at 40 °C. 10 mM tributylamine in 5% (v/v) methanol (A) and methanol (B) were used as mobile phase solvents. The gradient was set up as follows: 0 to 1 min, 95 to 85% A; 1 to 6 min, 65 to 40% A; 6 to 7 min, 40 to 0% A; 7 to 8 min, 0% A; 8 to 9 min, 100% A, at a flow rate of 0.3 mL min–1. The mass-spectra acquisition setup included negative mode electrospray ionization mode, source temperature of 120 °C, and desolvation temperature of 350 °C. Nitrogen was used as a sheath and auxiliary gas and collision gas (argon) was set to 1.1 mTorr. Gas flow for the desolvation and cone was set to 800 and 50 L/h, respectively. The scan time was 0.1 ms. The characteristic fragment ions used for measuring metabolites are detailed in *SI Appendix*, Table S1.

#### Measurement of pyruvate by gas chromatography-mass spectrometry (GC–MS).

Measurements were conducted as described by Xu et al. (53). The process was initiated by derivatizing pyruvate with the addition of methoxyamine hydrochloride dissolved in dry pyridine. The mixture was kept at 60 °C for 15 min, then cooled for 10 min. Subsequently, it was subjected to silylation by introducing N-tert-butyldimethylasyl-N-methyl-trifluoroacetamide (MTBSTFA) with 1% (w/v) tert-butylmethylchlorisilane (TBDMSCI), and kept at 60 °C overnight, resulting in silylated derivatives.

Pyruvate level in the samples were measured by an Agilent 7890 GC system (Agilent, Santa Clara, CA) coupled to an Agilent 5975C inert XL Mass Selective Detector (Agilent, Santa Clara, CA) with an autosampler (CTC PAL; Agilent, Santa Clara, CA). Metabolites were separated by an Agilent VF5ms GC column, 30 m × 0.25 mm × 0.25 m with a 10 m guard column (Part number: CP9013; Agilent, Santa Clara, CA). The oven temperature gradient was set as follows: 100 °C (held for 4 min), increased by 5 °C/min to 200 °C, then by 10 °C /min to 320 °C, and held at 320 °C for 10 min. Electron ionization (EI) is at 70 eV and the mass scan range was 100 to 600 amu. The ionization source temperature was set at 150 °C and the transfer line temperature at 300 °C. The characteristic fragment ions used for measuring metabolites are detailed in *SI Appendix*, Table S1.

### Measurement of Hormones by LC–MS/MS.

Frozen leaf tissue was ground into a fine powder using a tissue homogenizer. They were extracted using 4:1 methanol/ MilliQ water (v/v) added with butylated hydroxytoluene and formic acid. SA, 12OH-JA, JA d-5, ABA d-6,12OH-JA-Ile, IAA-13C6 were used as standards. Samples were quantified by Acquity TQD Tandem Quadrupole Mass Spectrometer with an Acquity BEH Amide column 1.7 µm × 2.1 mm × 50 mm (Acquity Group) with an autosampler (2777C Waters) as described by Bellucci et al. (20). An Acquity UPLC BEH C18 column (2.1 × 50 mm, 1.7 µm) was used on a Xevo TQ-XS mass spectrometer to separate the hormones. The separation employed a multistep gradient with mobile phase A (water + 0.1% formic acid) and mobile phase B (acetonitrile): 0 to 0.5 min, 95% A; 0.5 to 10 min, 95 to 70% A; 10 to 11 min, 70 to 5% A; 11 to 13 min, 5% A; 13 to 13.01 min, 5 to 95% A; 13.01 to 15 min, 95% A, at a flow rate of 0.5 mL/min. The column temperature was maintained at 40 °C, with source and desolvation temperatures set to 150 °C and 400 °C, respectively.

### Analysis of LC–MS/MS and GC–MS Data.

LC–MS/MS data were acquired using the MassLynx 4.0 (Waters Corporation, Milford, MA), while GC–MS data were acquired using the Agilent GC/MSD Chemstation (Agilent, Santa Clara, CA). Authentic standards, as mentioned above, were used to make external standard curves. The compounds were identified by their retention time and mass to charge (m/z) ratio with authentic standards. For both LC–MS and GC–MS datasets, conversion to MassLynx format was carried out, and subsequent data processing, including peak detection and quantification, was performed using QuanLynx software. Absolute quantification was achieved by employing an external standard curve that was normalized with internal standards.

### Plasmid Construction, Transformation, and Purification of GmISPS Protein.

The coding sequences of *EgISPS*, *TPS8*, and *TPS23* were modified by putting restriction enzyme sequences for BamH1 and Blp1, and Strep-SUMO tag under T promoter and a terminator. The desired sequences were inserted in a plasmid pET11a with the help of GenScript. *E. coli* BL21 (DE3) cells were transformed with the pET11a/ISPS plasmid constructs containing an N-terminal Strep-SUMO tag known to facilitate protein solubilization. A colony was inoculated in 5 mL LB with 50 mg/mL ampicillin and grown in a 37 °C shaker at 225 rpm overnight until they reached saturation (OD_600_ ≥2). 5 mL of this starter culture was added to 1 L LB with 50 mg/mL ampicillin and grown in a 37 °C shaker until it reached an OD_600_ of 0.6. Then IPTG was to a final concentration of 0.5 mM to induce the cultures and incubated overnight at 18 °C. The overnight grown culture of *E. coli* BL21 expressing the Strep-SUMO-tagged proteins was harvested by centrifugation at 5,000×*g* for 20 min at 4 °C, and the cell pellet was resuspended (50 mM Tris pH 8.0, 200 mM NaCl, 5 mM KCl, 50 mM MgCl_2_, 5% glycerol, 0.02% sodium azide, 0.5 mM phenylmethylsulfonyl fluoride, 1 mM tris(2-carboxyethyl) phosphine) to a density of 0.5 g mL^−1^. 1× Sigmafast® protease inhibitor, 0.1 mg mL^−1^ lysozyme and 1 mg mL^−1^ DNase (Sigma Aldrich, St. Louis, MO) was added to the resuspended cell pellet. Then the cells were lysed twice by passing them through a French Press at 104 psi.

The cell lysate was centrifuged at 40,000×*g* for 45 min at 4 °C to separate the soluble and insoluble fractions. The soluble fraction (supernatant) was filtered through a 0.22 mm syringe filter (Milipore Sigma, Burlington, MA). A 5 mL StrepTrap column (General Electric, Boston, MA) was used to purify the Strep-SUMO tagged EgISPS and GmISPS proteins. The column was first washed with buffer B before loading the sample. The protein was eluted using buffer B with 2.5 mM desthiobiotin (Sigma Aldrich, St. Louis, MO) and the protein peaks were identified using Fast protein liquid chromatography (FPLC). To cleave the Strep-SUMO tag, the eluted protein was incubated with Thermo Fisher SUMO protease overnight at 4 °C (Waltham, MA). Protein purity was determined by SDS-PAGE following the manufacturer’s protocol (Bio-Rad Bulletin 6201). Protein concentration was measured using the Bradford assay kit (Sigma-Aldrich, USA).

### RNA Extraction and Expression Analysis of *TPS8* and *TPS23*.

Soybean leaves were wounded using a forceps and the undamaged part of the leaves were collected in liquid nitrogen 10 min after wounding. The collected control and wounded leaves were subjected for RNA extraction using the RNeasy Mini Kit (Qiagen, Hilden, Germany) following the Company’s instruction. The synthesis of cDNA and qRT-PCR analysis were carried out following the protocols published by Santiago et al. (55). Primer-specific oligonucleotide sequences for qRT-PCR are provided in *SI Appendix*, Table S2. The acquired data were normalized against *GmActin* to obtain transcript levels of *TPS8* and *TPS23*.

### Quantification of ISPS Activity.

DMADP was purchased from Echelon Biosciences and its stock solution was prepared in 2 mM ammonium bicarbonate, pH 9.5. Assays were performed as described by Weraduwage et al (56). 5 µL of protein extract was mixed with assay buffer containing 50 mM HEPES pH 8, 10 mM MgCl_2_, 20 mM KCl, 2 mM DTT, 1 mM EDTA, 10% glycerol v/v, and 1.0 mg/mL BSA. DMADP was added to achieve final concentrations. Water was added to a total reaction volume of 300 µL. Reactions were done in 2 mL crimp top glass vials (Supelco, PA). After mixing all the reagents, the lids were crimp sealed, and the vials were incubated in a 40 °C water bath for 15 min. Then 1 mL of the headspace sample was collected in a syringe while putting in 1 mL water into the vial to prevent vacuum formation. The sample was then injected into the FIS to measure isoprene emission in the gas phase following the equation reported by Sahu et al. (49).

### Quantification of the Levels of Hydrogen Peroxide (H_2_O_2_).

Extraction and estimation of H_2_O_2_ levels were carried out using the Amplex Red Assay kit (Amplex Red, DMSO, Horseradish Peroxidase, and 5X phosphate buffer) following the protocol reported by Chakraborty et al. (57).

### Measurement of Isoprene Emission from Soybean Plants after Exposure to Heat Stress.

Soybean plants were grown and cultivated in the greenhouse as mentioned above. 60-d-old soybean plants were used to test the isoprene emission under heat stress. Isoprene emission was tested in two different setups using growth chambers and a LI-COR 6800 connected to a FIS. Soybean plants were transferred to two identical growth chambers equilibrated with 25 °C, 65% relative humidity, and 100% light intensity. Plants were allowed to acclimate to the growth chamber for 24 h. Mature leaves from different trifoliates were placed inside Teflon bags and kept closed with clips. The temperature was raised to 30 °C for 2 h and 40 °C for 24 h in one growth chamber. The air trapped inside the Teflon bags were collected using a 100 mL glass syringe at after 0, 2, and 24 h. The collected air was immediately injected into a FIS and isoprene level was recorded and calculated against a 3.22 µmol mol^−1^ isoprene standard.

For measuring isoprene emission using a LI-COR 6800 set up, the mature soybean leaf was placed in an LI-COR chamber, which was connected to a FIS as described above. Once photosynthesis stabilized at 30 °C, the temperature was raised to 38 °C and isoprene emission was measured by the FIS. The temperature was set back to 30 °C and the change in isoprene emission was also recorded by the FIS. A 3.22 µmol mol^−1^ isoprene standard was manually injected into the FIS for calculation of isoprene emission from the soybean leaves (49, 56).

### Statistical Analyses.

Student’s *t* test was used to evaluate pairwise comparisons of mean differences in TPS gene expression levels pre- and postwounding and wounding-induced change in H2O2 levels. ANOVA and Tukey’s honestly significant difference (HSD) mean separation test were used to evaluate differences in metabolite and hormone levels before, during, and after wounding/burning. ANOVA and Tukey’s HSD test were also used to determine differences in isoprene emission under high temperature and high CO_2_ conditions.

## Supplementary Material

Appendix 01 (PDF)

Movie S1.

## Data Availability

All data have been deposited in Dryad https://doi.org/10.5061/dryad.51c59zwkh (58).

## References

[1] T. D. Sharkey, A. E. Wiberley, A. R. Donohue, Isoprene emission from plants: Why and how. Ann. Bot. **101**, 5–18 (2008). 17921528 10.1093/aob/mcm240 PMC2701830

[2] K. Sindelarova , Global data set of biogenic VOC emissions calculated by the MEGAN model over the last 30 years. Atmos. Chem. Phys. **14**, 9317–9341 (2014).

[3] A. T. Lantz, J. Allman, S. M. Weraduwage, T. D. Sharkey, Isoprene: New insights into the control of emission and mediation of stress tolerance by gene expression. Plant Cell Environ. **42**, 2808–2826 (2019). 31350912 10.1111/pce.13629 PMC6788959

[4] J. M. Wedow, E. A. Ainsworth, S. Li, Plant biochemistry influences tropospheric ozone formation, destruction, deposition, and response. Trends Biochem. Sci. **46**, 992–1002 (2021). 34303585 10.1016/j.tibs.2021.06.007

[5] M. Bellucci, V. Locato, T. D. Sharkey, L. De Gara, F. Loreto, Isoprene emission by plants in polluted environments. J. Plant Interac. **18**, 2266463 (2023).

[6] M. Trainer , Models and observations of the impact of natural hydrocarbons on rural ozone. Nature **329**, 705–707 (1987).

[7] T. D. Sharkey, D. W. Gray, H. K. Pell, S. R. Breneman, L. Topper, Isoprene synthase genes form a monophyletic clade of acyclic terpene synthases in the Tps-b terpene synthase family. Evolution **67**, 1026–1040 (2013). 23550753 10.1111/evo.12013

[8] A. T. Lantz , Biochemical characterization of an isoprene synthase from Campylopus introflexus (heath star moss). Plant Physiol. Biochem. **94**, 209–215 (2015). 26113160 10.1016/j.plaphy.2015.06.008

[9] D. T. Hanson, S. Swanson, L. E. Graham, T. D. Sharkey, Evolutionary significance of isopreneemission from mosses. Am. J. Bot. **86**, 634–639 (1999). 10330065

[10] D. T. Tingey, R. C. Evans, E. H. Bates, M. L. Gumpertz, Isoprene emissions and photosynthesis in three ferns–the influence of light and temperature. Physiol. plant. **69**, 609–616 (1987).

[11] A. T. Lantz , Biochemical characterization of an isoprene synthase from *Campylopus introflexus* (heath star moss). Plant Physiol. Biochem. **94**, 209–215 (2015). 26113160 10.1016/j.plaphy.2015.06.008

[12] X. Yi , Genome assembly of the JD17 soybean provides a new reference genome for comparative genomics. G3 (Bethesda) **12**, jkac017 (2022). 35188189 10.1093/g3journal/jkac017 PMC8982393

[13] K. Behnke , Transgenic, non-isoprene emitting poplars don’t like it hot. Plant J. **51**, 485–499 (2007). 17587235 10.1111/j.1365-313X.2007.03157.x

[14] K. Behnke , RNAi-mediated suppression of isoprene emission in poplar transiently impacts phenolic metabolism under high temperature and high light intensities: A transcriptomic and metabolomic analysis. Plant Mol. Biol. **74**, 61–75 (2010). 20526857 10.1007/s11103-010-9654-z PMC3128716

[15] T. D. Sharkey, X. Y. Chen, S. Yeh, Isoprene increases thermotolerance of fosmidomycin-fed leaves. Plant Physiol. **125**, 2001–2006 (2001). 11299379 10.1104/pp.125.4.2001 PMC88855

[16] K. Sasaki , Plants utilize isoprene emission as a thermotolerance mechanism. Plant Cell Physiol. **48**, 1254–1262 (2007). 17711876 10.1093/pcp/pcm104

[17] V. Velikova, F. Loreto, T. Tsonev, F. Brilli, A. Edreva, Isoprene prevents the negative consequences of high temperature stress in Platanus orientalis leaves. Funct. Plant Biol. **33**, 931–940 (2006). 32689303 10.1071/FP06058

[18] V. Velikova , Increased thermostability of thylakoid membranes in isoprene-emitting leaves probed with three biophysical techniques. Plant Physiol. **157**, 905–916 (2011). 21807886 10.1104/pp.111.182519 PMC3192565

[19] V. Velikova, F. Loreto, On the relationship between isoprene emission and thermotolerance in *Phragmites australis* leaves exposed to high temperatures and during the recovery from a heat stress. Plant, Cell Environ. **28**, 318–327 (2005).

[20] M. Bellucci , The effect of constitutive root isoprene emission on root phenotype and physiology under control and salt stress conditions. Plant Direct **8**, e617 (2024). 38973810 10.1002/pld3.617 PMC11227114

[21] C. E. Vickers , Isoprene synthesis protects transgenic tobacco plants from oxidative stress. Plant, Cell Environ. **32**, 520–531 (2009). 19183288 10.1111/j.1365-3040.2009.01946.x

[22] F. Loreto , Ozone quenching properties of isoprene and its antioxidant role in leaves. Plant Physiol. **126**, 993–1000 (2001). 11457950 10.1104/pp.126.3.993 PMC116456

[23] F. Loreto, V. Velikova, Isoprene produced by leaves protects the photosynthetic apparatus against ozone damage, quenches ozone products, and reduces lipid peroxidation of cellular membranes. Plant Physiol. **127**, 1781–1787 (2001). 11743121 PMC133581

[24] M. Loivamäki, R. Mumm, M. Dicke, J.-P. Schnitzler, Isoprene interferes with the attraction of bodyguards by herbaceous plants. Proc. Natl. Acad. Sci. U.S.A. **105**, 17430–17435 (2008). 18987312 10.1073/pnas.0804488105 PMC2582323

[25] J. Laothawornkitkul , Isoprene emissions influence herbivore feeding decisions. Plant Cell Environ. **31**, 1410–1415 (2008). 18643955 10.1111/j.1365-3040.2008.01849.x

[26] A. Sahu , Isoprene deters insect herbivory by priming plant hormone response. Sci. Adv. **11**, eadu4637 (2025), 10.1126/sciadv.adu4637, in press. 40249816 PMC12007590

[27] T. D. Sharkey, F. Loreto, Water stress, temperature, and light effects on the capacity for isoprene emission and photosynthesis of kudzu leaves. Oecologia **95**, 328–333 (1993). 28314006 10.1007/BF00320984

[28] E. Vanzo , Facing the future: Effects of short-term climate extremes on isoprene-emitting and nonemitting poplar. Plant Physiol. **169**, 560–575 (2015). 26162427 10.1104/pp.15.00871 PMC4577423

[29] Z. Zuo , Isoprene acts as a signaling molecule in gene networks important for stress responses and plant growth. Plant Physiol. **180**, 124–152 (2019). 30760638 10.1104/pp.18.01391 PMC6501071

[30] K. G. S. Dani, F. Loreto, Plant volatiles as regulators of hormone homeostasis. New Phytol. **234**, 804–812 (2022). 35170033 10.1111/nph.18035

[31] K. G. S. Dani , Isoprene enhances leaf cytokinin metabolism and induces early senescence. New Phytol. **234**, 961–974 (2022). 34716577 10.1111/nph.17833 PMC9300082

[32] S. Parveen , Plant hormone effects on isoprene emission from tropical tree in. Plant, Cell Environ. **42**, 1715–1728 (2019). 30610754 10.1111/pce.13513

[33] S. M. Weraduwage, A. Sahu, M. Kulke, J. V. Vermaas, T. D. Sharkey, Characterization of promoter elements of isoprene-responsive genes and the ability of isoprene to bind START domain transcription factors. Plant Direct **7**, e483 (2023). 36742092 10.1002/pld3.483 PMC9889695

[34] S. M. Weraduwage , The isoprene-responsive phosphoproteome provides new insights into the putative signaling pathways and novel roles of isoprene. Plant Cell Environ. **47**, 1099–1117 (2024). 38038355 10.1111/pce.14776

[35] S. Poudel , Quantifying the physiological, yield, and quality plasticity of southern USA soybeans under heat stress. Plant Stress **9**, 100195 (2023).

[36] A. S. Huseth , Current distribution and population persistence of five lepidopteran pests in U.S. soybean. J. Integr. Pest Manage. **12**, 11 (2021).

[37] R. K. Monson, R. T. Jones, T. N. Rosenstiel, J. P. Schnitzler, Why only some plants emit isoprene. Plant Cell Environ. **36**, 503–516 (2013). 22998549 10.1111/pce.12015

[38] R. K. Monson, R. T. Jones, T. N. Rosenstiel, J. P. Schnitzler, Why only some plants emit isoprene. Plant Cell Environ **36**, 503–516 (2013). 22998549 10.1111/pce.12015

[39] T. D. Sharkey , Evolution of the isoprene biosynthetic pathway in kudzu. Plant Physiol. **137**, 700–712 (2005). 15653811 10.1104/pp.104.054445 PMC1065370

[40] M. Köksal, I. Zimmer, J. P. Schnitzler, D. W. Christianson, Structure of isoprene synthase illuminates the chemical mechanism of teragram atmospheric carbon emission. J. Mol. Biol. **402**, 363–373 (2010). 20624401 10.1016/j.jmb.2010.07.009 PMC2942996

[41] D. W. Gray, S. R. Breneman, L. A. Topper, T. D. Sharkey, Biochemical characterization and homology modeling of methylbutenol synthase and implications for understanding hemiterpene synthase evolution in plants*. J. Biol. Chem. **286**, 20582–20590 (2011). 21504898 10.1074/jbc.M111.237438 PMC3121459

[42] J. P. Schnitzler , Biochemical properties of isoprene synthase in poplar (*Populus x canescens*). Planta **222**, 777–786 (2005). 16052321 10.1007/s00425-005-0022-1

[43] F. Loreto, T. D. Sharkey, Isoprene emission by plants is affected by transmissible wound signals. Plant, Cell Environ. **16**, 563–570 (1993).

[44] K. Kim, A. R. Portis, Oxygen-dependent H2O2 production by Rubisco. FEBS Lett. **571**, 124–128 (2004). 15280029 10.1016/j.febslet.2004.06.064

[45] Y. Liu , Role of hydrogen peroxide in regulating glucose-6-phosphate dehydrogenase activity under salt stress. Biol. Plant. **56**, 313–320 (2012).

[46] V. J. Streusand, A. R. Portis, “Effects of 6-Phosphogluconate and Rubp on Rubisco Activation State and Activity” in Progress in Photosynthesis Research: Volume 3 Proceedings of the VIIth International Congress on Photosynthesis Providence, Rhode Island, USA, August 10–15, 1986, J. Biggins, Ed., (Springer Netherlands, Dordrecht, 1987), 10.1007/978-94-017-0516-5_79, pp. 383–386.

[47] C. C. Burke, M. R. Wildung, R. Croteau, Geranyl diphosphate synthase: Cloning, expression, and characterization of this prenyltransferase as a heterodimer. Proc. Natl. Acad. Sci. U.S.A. **96**, 13062–13067 (1999). 10557273 10.1073/pnas.96.23.13062 PMC23900

[48] A. Takaya , Cloning, expression and characterization of a functional cDNA clone encoding geranylgeranyl diphosphate synthase of Hevea brasiliensis. Biochim. Biophys. Acta **1625**, 214–220 (2003). 12531482 10.1016/s0167-4781(02)00602-4

[49] A. Sahu, M. G. Mostofa, S. M. Weraduwage, T. D. Sharkey, Hydroxymethylbutenyl diphosphate accumulation reveals MEP pathway regulation for high CO2-induced suppression of isoprene emission. Proc. Natl. Acad. Sci. U.S.A. **120**, e2309536120 (2023). 37782800 10.1073/pnas.2309536120 PMC10576107

[50] A. J. Saathoff, J. Welles, Gas exchange measurements in the unsteady state. Plant, Cell Environ. **44**, 3509–3523 (2021). 34480484 10.1111/pce.14178 PMC9292621

[51] A. M. McClain, J. A. Cruz, D. M. Kramer, T. D. Sharkey, The time course of acclimation to the stress of triose phosphate use limitation. Plant Cell Environ. **46**, 64–75 (2023). 36305484 10.1111/pce.14476 PMC10100259

[52] T. D. Sharkey, What gas exchange data can tell us about photosynthesis?. Plant Cell Environ. **39**, 1161–1163 (2016). 26390237 10.1111/pce.12641

[53] Y. Xu , The metabolic origins of non-photorespiratory CO_2_ release during photosynthesis: A metabolic flux analysis. Plant Physiol. **186**, 297–314 (2021). 33591309 10.1093/plphys/kiab076 PMC8154043

[54] Y. Xu, T. Wieloch, J. A. M. Kaste, Y. Shachar-Hill, T. D. Sharkey, Reimport of carbon from cytosolic and vacuolar sugar pools into the Calvin–Benson cycle explains photosynthesis labeling anomalies. Proc. Natl. Acad. Sci. U.S.A. **119**, e2121531119 (2022). 35259011 10.1073/pnas.2121531119 PMC8931376

[55] J. P. Santiago, T. D. Sharkey, Pollen development at high temperature and role of carbon and nitrogen metabolites. Plant Cell Environ. **42**, 2759–2775 (2019). 31077385 10.1111/pce.13576

[56] S. M. Weraduwage, B. Rasulov, A. Sahu, Ü. Niinemets, T. D. Sharkey, “Chapter Eight - Isoprene measurements to assess plant hydrocarbon emissions and the methylerythritol pathway” in Methods in Enzymology, J. Jez, Ed. (Academic Press, 2022), **vol. 676**, pp. 211–237. 10.1016/bs.mie.2022.07.020 36280351

[57] S. Chakraborty , Quantification of hydrogen peroxide in plant tissues using Amplex Red. Methods **109**, 105–113 (2016). 27476009 10.1016/j.ymeth.2016.07.016

[58] M. G. Mostofa , Data from “Cryptic isoprene emission of soybeans.” Dryad. 10.5061/dryad.51c59zwkh. Accessed 24 May 2025. PMC12184331 40504154

