# Supplementary material for: Cryptic isoprene emission of soybeans
Source: Proc Natl Acad Sci U S A. 2025 Jun 12;122(24):e2502360122. doi: 10.1073/pnas.2502360122 (PMC12184331; doi:10.1073/pnas.2502360122)
Supplement: Supplementary file 1 — Appendix 01 (PDF) [file pnas.2502360122.sapp.pdf]

Supplemental figures and tables for

Cryptic isoprene emission of soybeans

Mohammad Golam Mostofa[1,2,3,4,*], Abira Sahu[1,3,5,] Yuan Xu[1], Insiya Basrai[2], Lior Doron[1], Violet Lefrancois[2], and Thomas D. Sharkey[1,2,3,*]

[1]Department of Energy Plant Research Laboratory, Michigan State University, East Lansing, Michigan, USA

[2]Department of Biochemistry and Molecular Biology, Michigan State University, East Lansing, MI, USA,

[3]Plant Resilience Institute, Michigan State University, East Lansing, MI, USA.

[4]Department of Chemistry, State University of New York College of Environmental Science and Forestry, Syracuse, NY, USA

[5]Department of Environmental Health Sciences, University of Alabama, Birmingham, Alabama, USA

Contents

Figure S1: An expanded version of the phylogenetic tree of *isoprene synthase* gene sequences and *E-β-ocimene synthase* gene sequences.
Figure S2: Alignment of isoprene synthase protein sequences of legumes with known isoprene emitters.
Figure S3: Plasmid transformation and purification steps for expressed proteins and *in-vitro* assay of isoprene production using purified proteins.
Figure S4: A LI-COR 6800 is clamped on one side of the mid rib of a soybean terminal leaflet.
Figure S5. Evidence of isoprene emission from the undamaged part of wounded soybean leaves.
Figure S6: *A/Ci* response curves in soybean leaves upon wounding.
Figure S7: *A/Ci* response curve in soybean leaves upon burning.
Figure S8: MEP pathway and central carbon metabolism-related metabolites in soybean leaves before (S1), during (S2), and after (S3) burning.
Figure S9: Hormonal profiles in soybean leaves before (S1), during (S2), and after (S3) wounding.

Table S1: List of primers used for quantitative real time PCR.
Table S2: Gas exchange parameters before, during, and after an isoprene burst.

Movie S1: Video showing isoprene emission and concurrent photosynthesis measurements from the undamaged part of wounded soybean leaves. The movie shows synchronous changes in photosynthesis-related parameters (LI-COR) and isoprene emission (Fast Isoprene Sensor, FIS) upon wounding of soybean leaves with a hot forceps. From zero to 22 s wounding, at 28 s

the view is moved to the LI-COR 6800. Stomatal conductance is shown in green while CO_2 is shown in purple. At 36 s the speed is increased from normal to 20 times normal speed. At 38 s stomatal conductance begins to rise while CP_2 assimilation falls. At 39 s the isoprene signal on the Fast Isoprene Sensor (on the left) begins to increase. At the end (approximately 12 min after wounding), isoprene has returned to background levels and both stomatal conductance and CO_2 assimilation are declining.

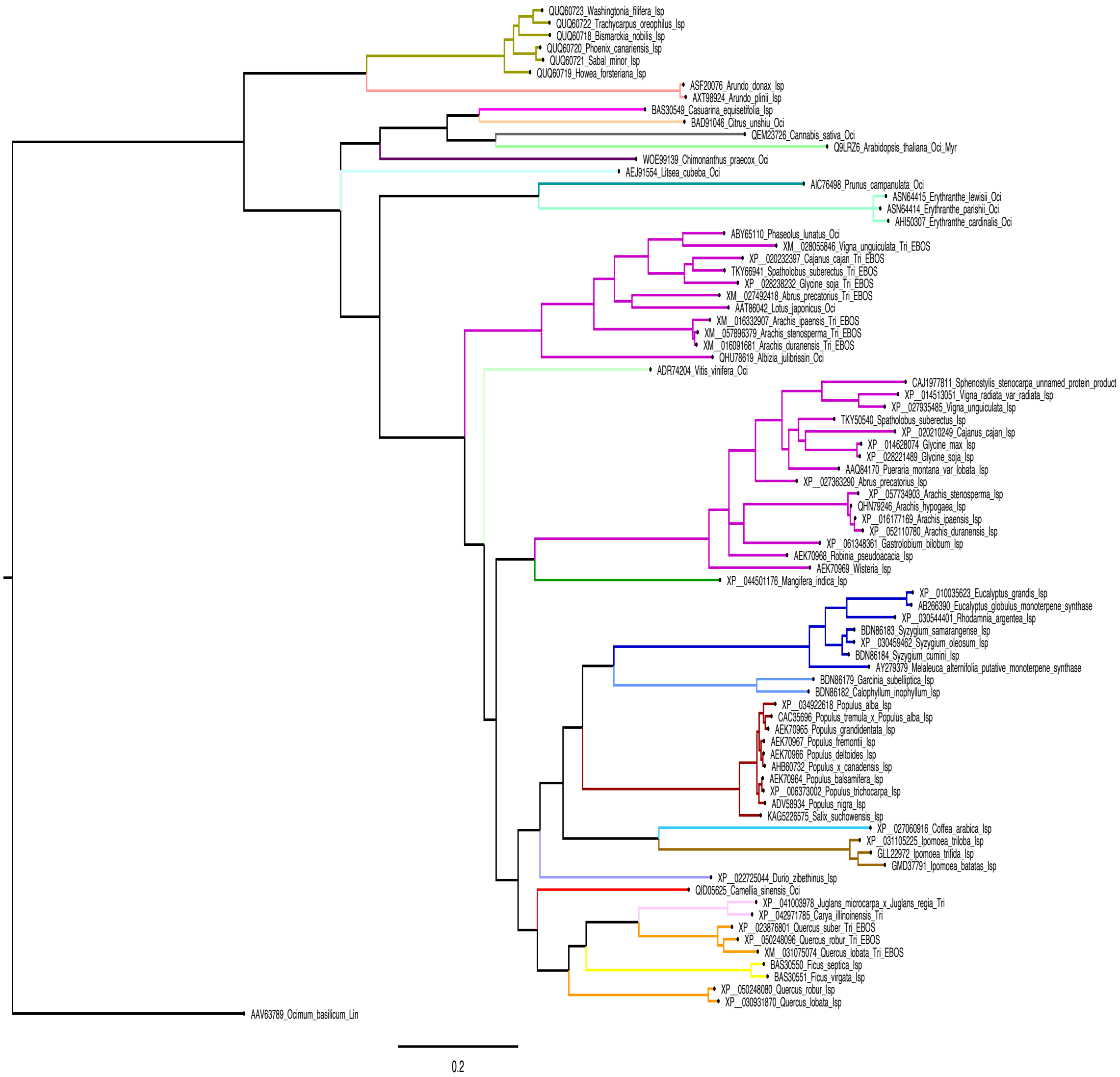

Figure S1: An expanded version of the phylogenetic tree of *isoprene synthase* gene sequences and *E-β-ocimene synthase* gene sequences. 1: IspS from Arecaceae (*Washingtonia, Trachycarpus, Bismarckia, Phoenix, Sabal, Howea*) and Poaceae (*Arundo*); 2: Oci from Rutaceae (*Citrus*), Cannabaceae (*Cannabis*), Brassicaceae (*Arabidopsis*), Calycanthaceae (*Chimonanthus*), ISPS from Casuarinaceae (*Casuarina*); 3: Oci from Phrymaceae (*Erythranthe*), Rosaceae (*Prunus*); 4: Oci from Fabaceae (*Glycine, Sphenostylis, Vigna, Spatholobus, Cajanus, Abrus, Robinia, Wisteria, Arachis, Phaseolus, Lotus, Albizia*); 5: ISPS from Fabaceae (*Glycine, Sphenostylis, Vigna, Spatholobus, Cajanus, Pueraria, Abrus, Robinia, Wisteria, Arachis, Gastrolobium*); 6: ISPS from Myrtaceae (*Eucalyptus, Rhodamnia, Syzygium, Melaleuca*), Clusiaceae (*Garcinia, Calophyllum*); 7: ISPS from Salicaceae (*Populus, Salix*); 8: ISPS from Rubiaceae (*Coffea*), Convolvulaceae (*Ipomoea*); 9: Oci from Juglandaceae (*Juglans*, *Carya*), Fagaceae (*Quercus*); 10: ISPS from Moraceae (*Ficus*); 11: ISPS from Fagaceae (*Quercus*). NCBI was searched for genes labeled isoprene synthase and ocimene synthase. Many genes previously identified as ocimene synthase are now labeled “tricyclene synthase EBOS”. We believe these to be E-β-ocimene genes and have included them here. The sequences were aligned in CLC Genomics Workbench, and the tree was estimated in MrBayes with 3 million generations using 82 total sequences. The final standard deviation

value was 0.00943. The resulting tree was drawn in FigTree (version 1.4.4).

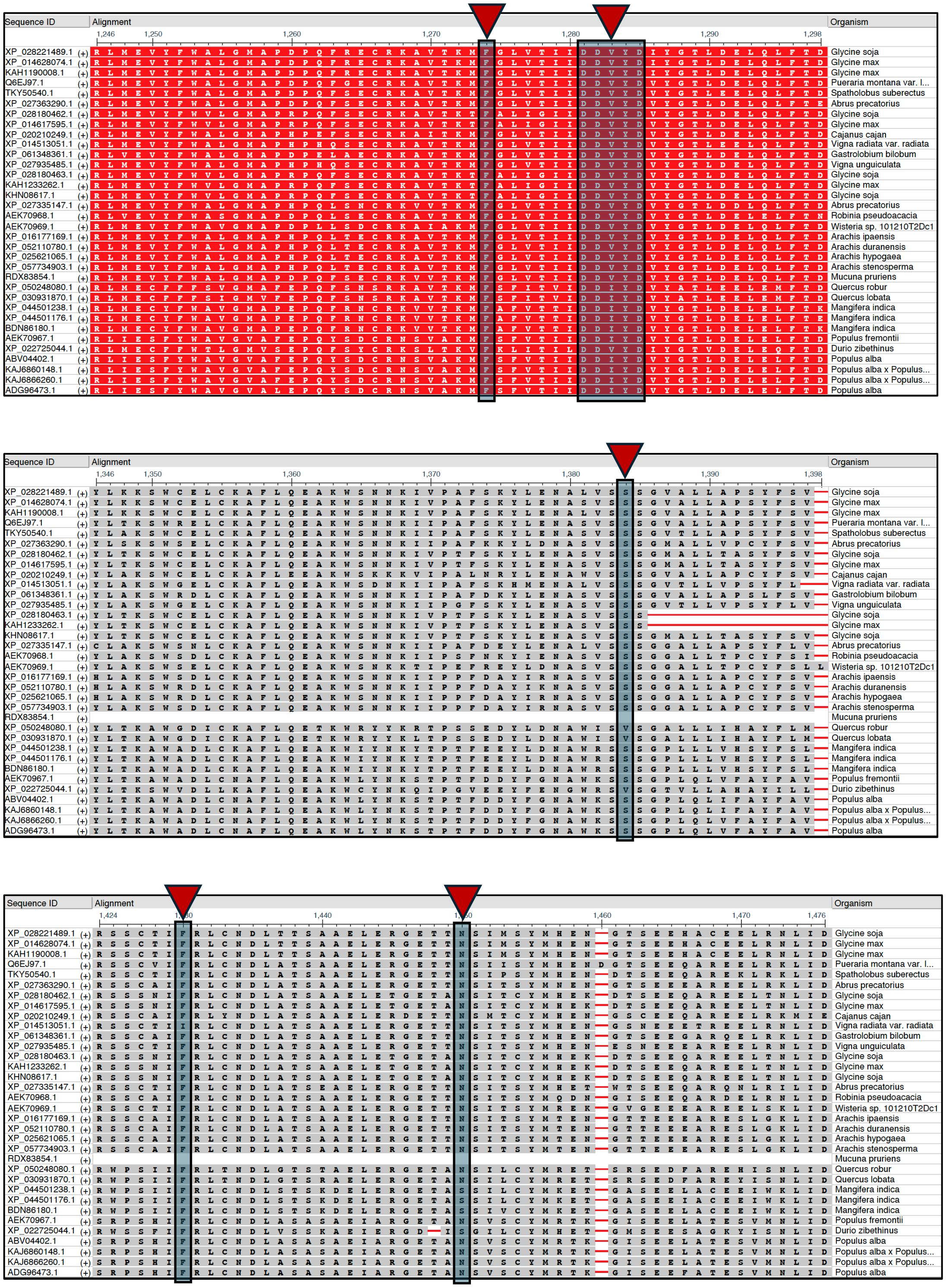

Figure S2: Alignment of isoprene synthase protein sequences of legumes with known isoprene emitters. Vertical shades highlight the variations in four unique amino acids

(phenylalanine, F; serine, S, and asparagine, N) and DDXXD conserved sequence across the species.

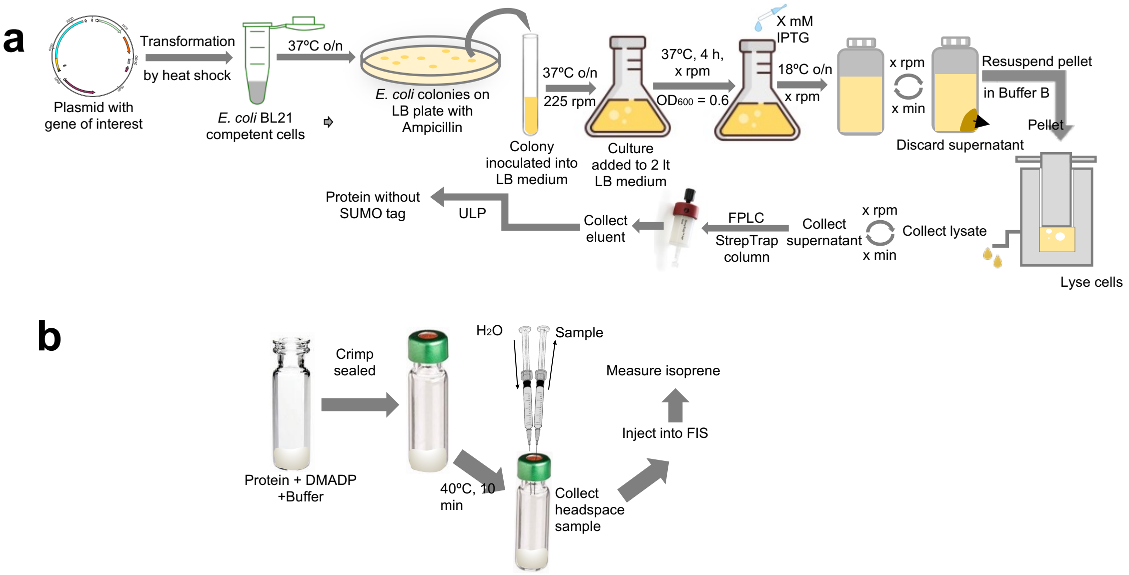

Figure S3: Plasmid transformation and purification steps for expressed proteins and *in-vitro* assay of isoprene production using purified proteins. (a) Representative plasmid constructed using genes of interest (*GmTPS8*, *GmTPS23*, and *EgISPS*) were transformed to *E.Coli* BL21 competent cells followed by lysis using French Press and purification by Fast protein liquid chromatography (FPLC). (b) Steps involved in measuring isoprene synthase activity of purified proteins using dimethylallyl diphosphate (DMADP) as substrate.

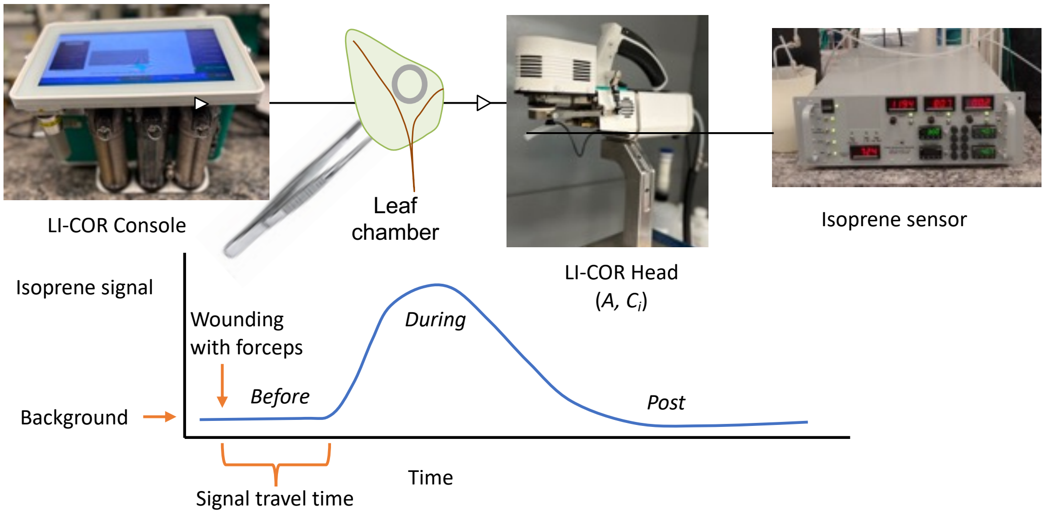

Figure S4: A LI-COR 6800 is clamped on one side of the mid rib of a soybean terminal leaflet. The other side, or a lateral and distal leaflet, is crushed with hot forceps or burned with a butane lighter. Air exiting the LI-COR head is directed to a Fast Isoprene Sensor. Isoprene emitted from the undamaged part of the leaves in parallel with gas exchange parameters like photosynthesis rate (*A*) and stomatal conductance (g_{sw}) is recoded in FIS and LI-COR, respectively.

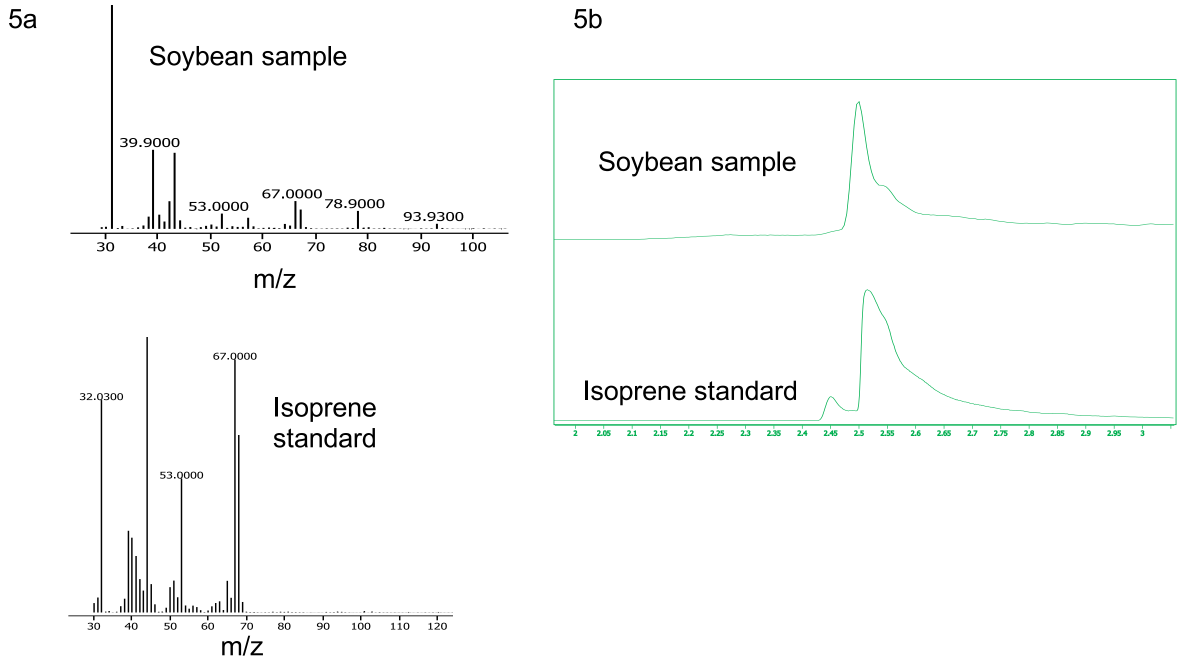

Figure S5. Evidence of isoprene emission from the undamaged part of wounded soybean leaves. Detection of isoprene peaks was carried out using Gas Chromatography-Solid Phase Microextraction (GC-SPME). Representative MS/MS spectra (molecular mass m/z 67, characteristic fragment ion for isoprene) (A) and GC-MS chromatograms (retention time 2-3 min) (B) represent the isoprene emission peaks from soybean leaf and isoprene standard (3 ppm).

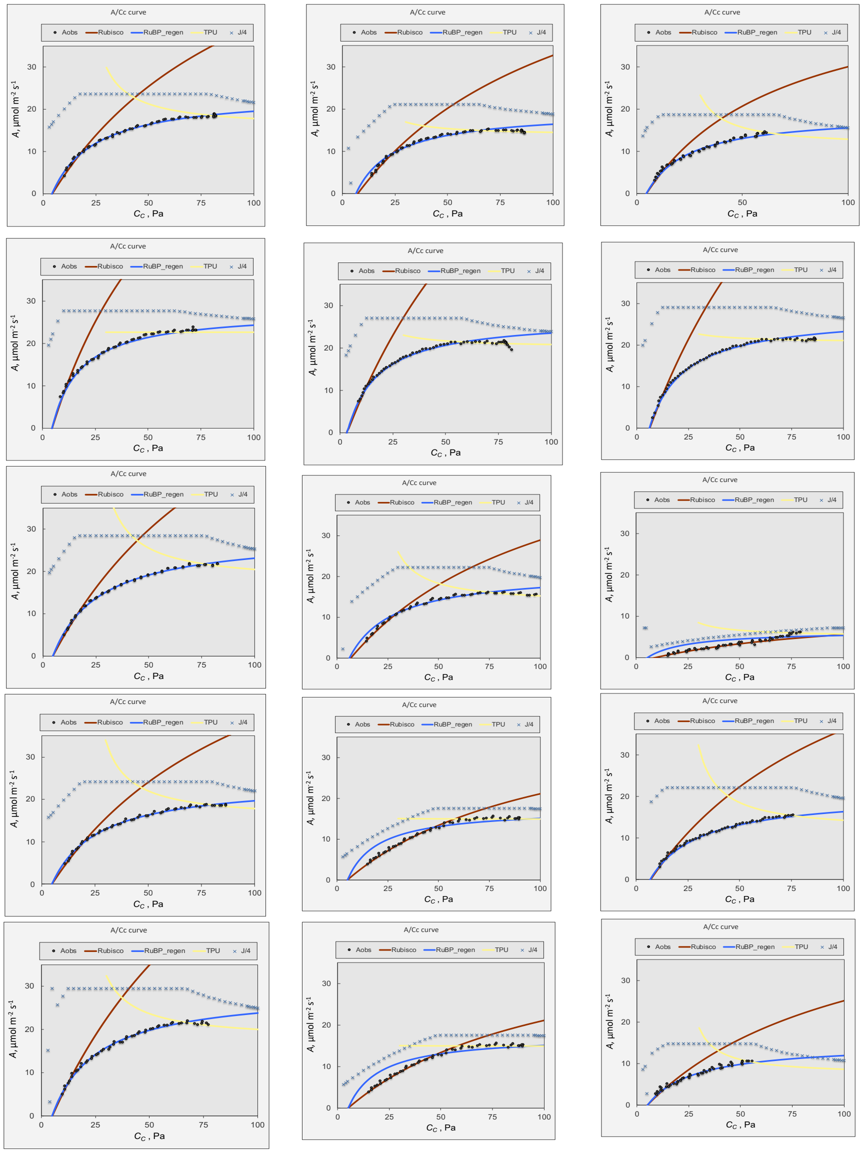

Figure S6: *A/Ci* response curves in soybean leaves upon wounding. CO_2 assimilation (*A*) rate is recorded at varying level of CO_2 (50 to 1000 µmol mol^{-1}) using dynamic assimilation technique (DAT) to identify dynamic changes in photosynthesis related parameters, including rubisco activity limitation, ribulose bisphosphate (RuBP) regeneration limitation, and triose phosphate utilization limitation in wounded soybean leaves. Assmilation data and stomatal conductance is used to generagte the CO_2 concentration at the sites of carboxylation, C_c.

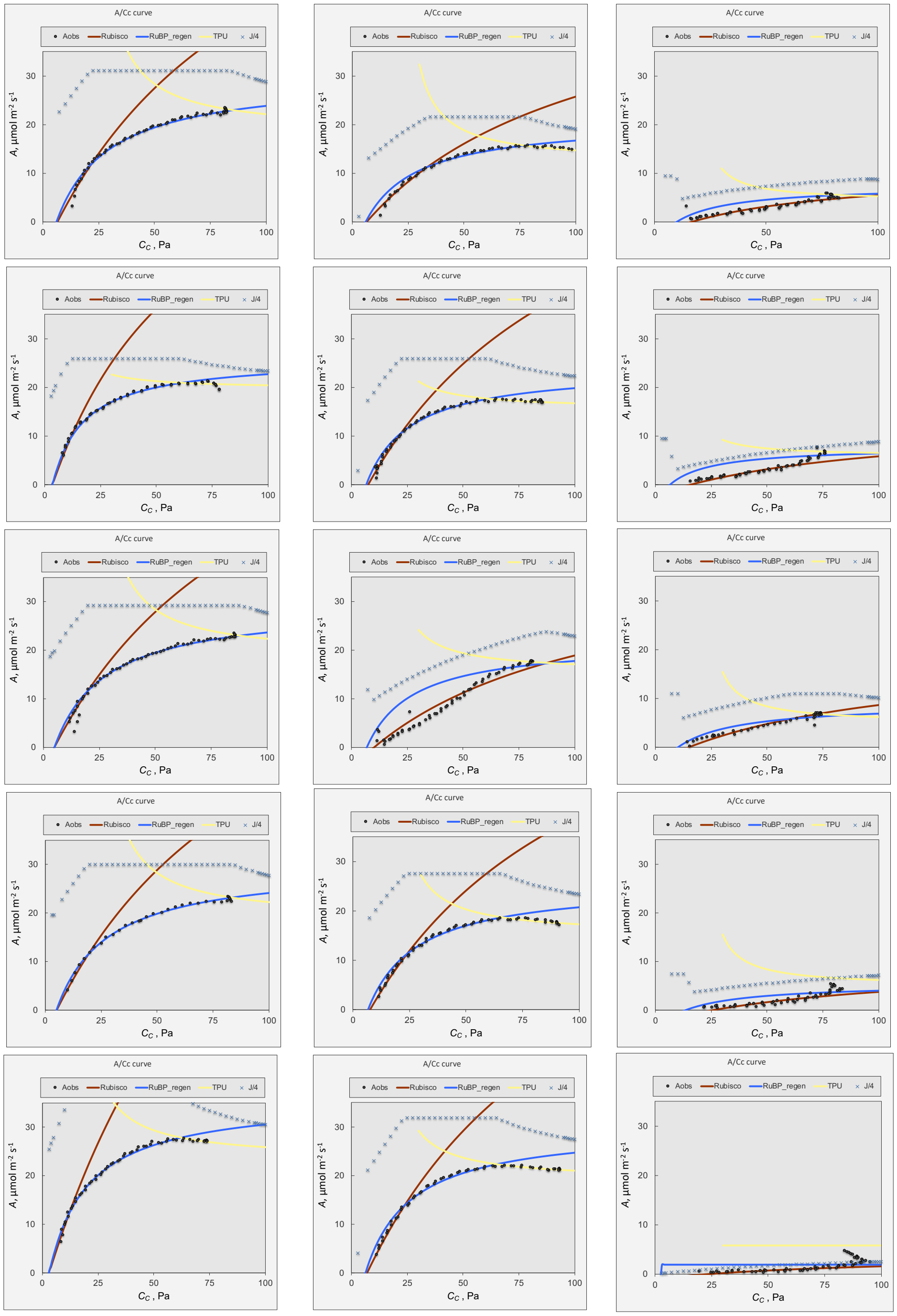

Figure S7: *A/Ci* response curve in soybean leaves upon burning. CO_2 assimilation (*A*) rate is recorded at varying level of CO_2 (50 to 1000 ppm) using dynamic assimilation technique (DAT) to identify dynamic changes in photosynthesis related parameters, including rubisco activity limitation, ribulose bisphosphate (RuBP) regeneration limitation, and triose phosphate utilization limitation in burnt soybean leaves. Assmilation data and stomatal conductance is used to generagte the CO_2 concentration at the sites of carboxylation, C_c.

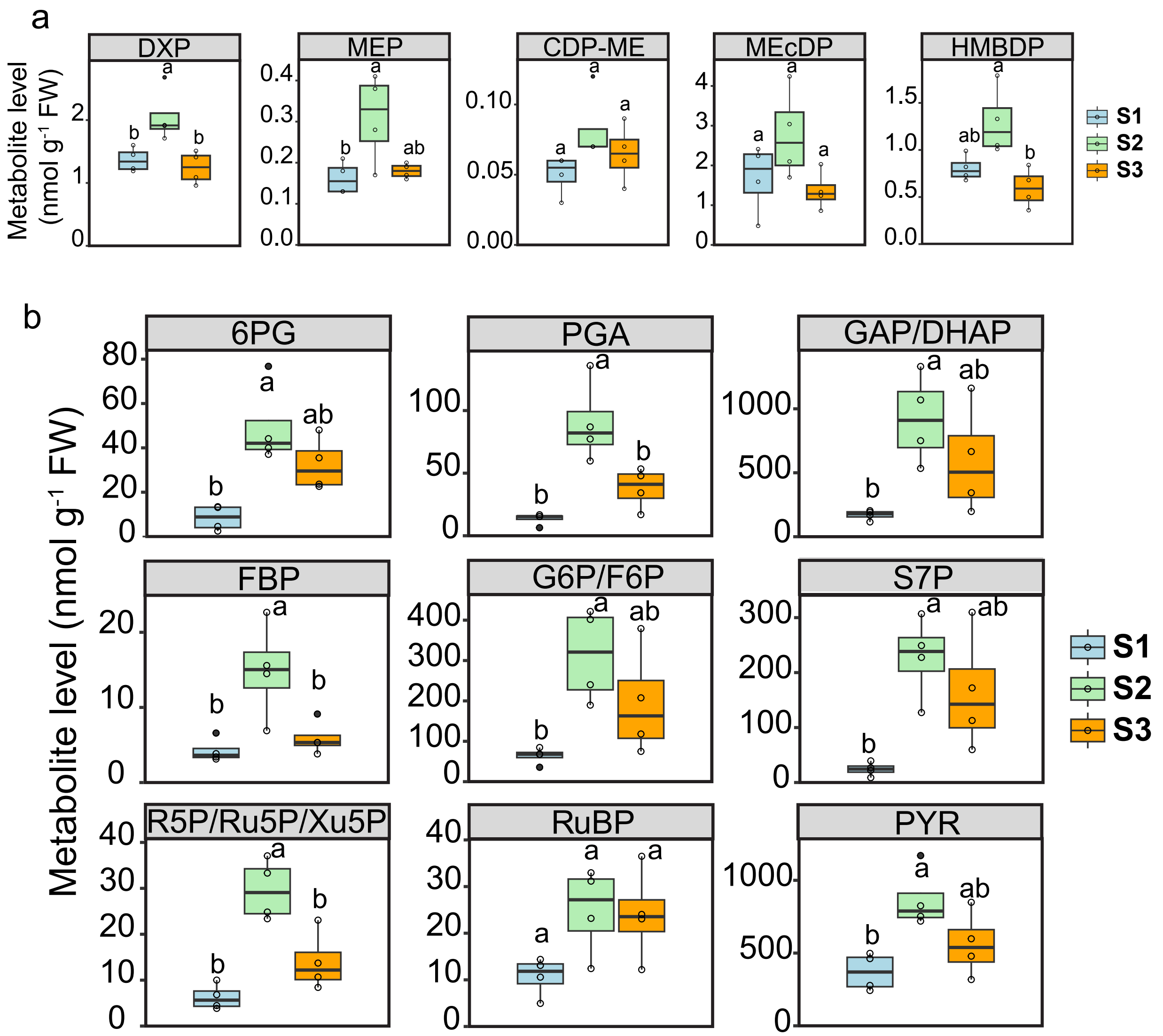

Figure S8: MEP pathway and central carbon metabolism-related metabolites in soybean leaves before (S1), during (S2), and after (S3) burning. (a) The levels of MEP pathway metabolites, including 1-deoxyxylulose 5-phosphate (DXP), 2-*C*-methyl-D-erythritol 4-phosphate (MEP), 4-diphosphocytidyl-2-*C*-methyl-D-erythritol (CDP-ME), 4-diphosphocytidyl-2-*C*-methyl-D-erythritol 2-phosphate (CDP-MEP), 2-*C*-methyl-D-erythritol 2,4-cyclodiphosphate (MEcDP), and 1-hydroxy-2-methyl-2-(*E*)-butenyl 4-diphosphate (HMBDP) and (b) central carbon metabolism metabolites, including pyruvate (PYR), glyceraldehyde 3-phosphate (GAP), 6-phosphogluconate (6PG), 3-hosphoglyceraldehyde (PGA), GAP/dihydroxyacetone phosphate (DHAP), fructose 6-bisphosphate (FBP)/glucose 6-phosphate (G6P), seduheptulose 7-phosphate (S7P), ribose 5-phosphate (R5P)/ Ribulose 5-phosphate (Ru5P)/xylulose 5-phosphate (Xu5P), rubisco bisphosphate (RuBP), and pyruvate (PYR) are measured in soybean leaf samples collected S1, S2, and S3 upon burning of soybean leaves. Different alphabetical letters (a and b) indicate significant differences among the treatment conditions at $p<0.05$ according to least significant difference (LSD). Statistically significant differences in MEP and CBC metabolites between S1, S2, and S3 were calculated by ANOVA and Tukey's HSD and are indicated by lowercase letters.

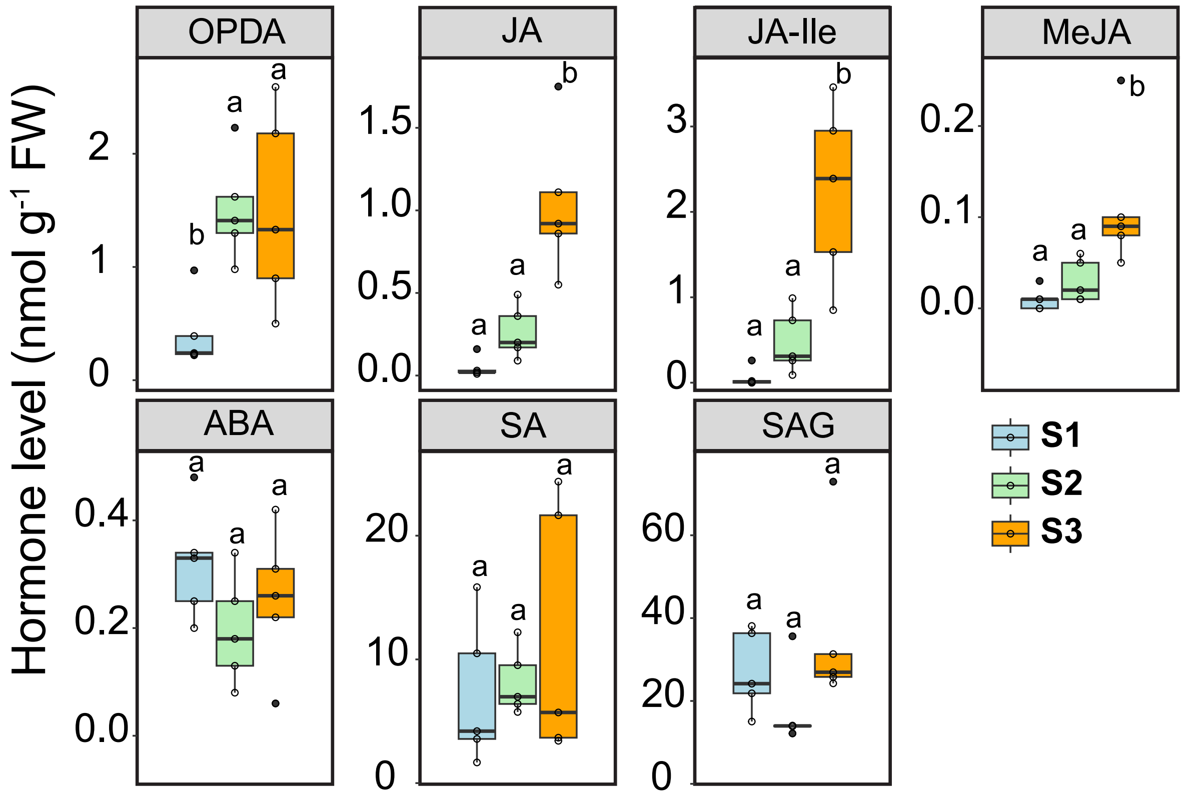

Figure S9: Hormonal profiles in soybean leaves before (S1), during (S2), and after (S3) wounding. Different alphabetical letters (a and b) indicate significant differences among the treatment conditions at $p<0.05$ according to least significant difference (LSD). OPDA, 12-oxo-phytodienoic acid; JA, jasmonic acid; JA-Ile, jasmonic acid isoleucine; MeJA, methyl jasmonate; ABA, abscisic acid; SA, salicylic acid; SAG, salicylate glucoside. Statistically significant differences in hormone levels between S1, S2, and S3 were calculated by ANOVA and Tukey's HSD and are indicated by lowercase letters.

Table S1. The characteristic fragment ions used for measuring metabolites. Parameters for transitions of measured metabolites in LC-MS/MS and GC-MS. Multiple reaction monitoring (MRM) is used for Ion-pair chromatography – tandem mass spectrometry (IPC-MS/MS), with a dwell time of 20 ms set for each transition. Q1, m/z of the precursor ion; Q3, m/z of the product ion. Cone and collision energy were optimized by direct infusion of standards. Selected ion monitoring (SIM) is for GC-MS. Pyruvate and norvaline were measured by GC-MS by tert-butyldimethylsilyl (TBDMS) derivatization.

**Multiple reaction monitoring (MRM) with LC-MS/MS**	
**Metabolites**	**$Q_1[Q_3]$ (m/z) of mass isotopomers**
**PGA**	185[97]
**GAP/DHAP**	169[97]
**R5P/RU5P/XU5P**	229[97]
**RUBP**	309[97]
**G6P/F6P**	259[97]
**6PG**	275[97]
**S7P**	289[97]
**FBP**	339[97]
**6PG**	275[97]
**DXP**	213[79]
**MEP**	215[79]
**CDP-ME**	520[322]
**MEcDP**	277[79]
**HMBDP**	261[79]
**IDP+DMADP**	245[79]
**OPDA**	291[165]
**JA**	209[59]
**JA-Ile**	322[130]
**MeJA**	225[151]
**ABA**	263[153]
**SAG**	299[137]
**SA**	137[73]
**Selected ion monitoring (SIM) with GC-MS**	
**Metabolite**	**Mass range**
**Pyruvate**	*174*

Table S2: List of primers used for quantitative real time PCR (qRT-PCR) for checking the expression of soybean isoprene synthase genes *TPS8* and *TPS23*. *Glycine max actin* (*Gmactin-11*) was used as reference gene for this study.

Gene	Primer
Gmactin-11-F	$^{5'}$GAGCTATGAATTGCCTGATGG$^{3'}$
Gmactin-11-R	$^{5'}$CGTTTCATGAATTCCAGTAGC$^{3'}$
TPS8-F	$^{5'}$AGCACCCTGAAAGCAAAAGG$^{3'}$
TPS8-R	$^{5'}$TGAGGAGGAAACGGATGCAT$^{3'}$
TPS23-F	$^{5'}$CAGTGTGCCAAGAACAGGAC$^{3'}$
TPS23-R	$^{5'}$ATGCTCCTCAGAAGTGCCAT$^{3'}$

Table S3: Gas exchange parameters before, during, and after an isoprene burst.

Wounding

Before	W1	W2	W3	W4	W5	Average	Stdev
V_{cmax}	82	107	97	78	115	**96**	16
J	95	111	114	97	118	**107**	10
**TPU**	5.2	7.5	5.8	5.1	6.0	**5.93**	0.99
R_d*	0.01	0.01	0.01	0.01	0.51	**0.11**	0.22
g_m*	1.27	1.13	1.90	1.86	1.96	**1.62**	0.39
α_G	0.18	0.00	0.05	0.08	0.06	**0.07**	0.07
α_S	0.61	0.00	0.71	0.69	0.55	**0.51**	0.29
**SSR**	5.28	4.95	2.27	3.51	5.97	**4.40**	1.49
**During**							
V_{cmax}	71	129	62	44	56	**72**	33
J	85	108	89	70	98	**90**	14
**TPU**	5.1	6.7	4.7	5.0	5.5	**5.41**	0.78
R_d*	1.43	0.01	0.93	0.03	2.00	**0.88**	0.87
g_m*	0.80	0.96	5.38	1.24	4.30	**2.54**	2.14
α_G	0.00	0.35	0.00	0.00	0.00	**0.07**	0.16
α_S	0.21	0.00	0.58	0.00	0.48	**0.26**	0.27
**SSR**	3.09	5.80	3.01	4.69	1.41	**3.60**	1.69
**After**			**				
V_{cmax}	51	141	13	78	52	**67**	48
J	75	116	29	88	59	**74**	33
**TPU**	3.7	7.4	1.9	4.4	2.4	**3.97**	2.16
R_d*	0.01	1.59	0.71	1.49	0.01	**0.76**	0.76
g_m*	0.55	1.51	30.00	1.63	0.42	**6.82**	12.97
α_G	0.00	0.00	0.34	0.00	0.00	**0.07**	0.15
α_S	0.75	0.11	0.49	0.75	0.75	**0.57**	0.28
**SSR**	6.97	2.66	2.71	3.22	9.91	**5.09**	3.24

Burning

Before	W1	W2	W3	W4	W5	W6	Average	Stdev
V_{cmax}	94	87	91	95	149	98	**102**	23
J	124	104	117	120	144	114	**121**	13
TPU	6.5	6.6	6.3	6.3	7.8	5.7	**6.53**	0.68
R_d*	1.21	0.01	0.01	0.14	0.01	2.00	**0.56**	0.85
g_m*	1.63	0.92	2.45	2.92	1.59	2.85	**2.06**	0.80
α_G	0.00	0.23	0.00	0.00	0.37	0.00	**0.10**	0.16
α_S	0.75	0.08	0.75	0.75	0.47	0.75	**0.59**	0.27
SSR	3.85	4.53	3.33	1.95	6.47	1.25	**3.56**	1.87

During			**					
V_{cmax}	55	88	43	86	102	53	**71**	24
J	87	104	95	110	128	81	**101**	17
TPU	4.3	5.8	5.8	5.7	7.0	3.9	**5.43**	1.13
R_d*	0.72	2.00	2.00	2.00	1.85	0.69	**1.54**	0.65
g_m*	29.94	0.89	1.18	1.59	2.32	5.79	**6.95**	11.40
α_G	0.00	0.00	0.00	0.00	0.00	0.00	**0.00**	0.00
α_S	0.75	0.31	0.41	0.51	0.41	0.75	**0.52**	0.19
SSR	3.09	6.00	90.00	6.49	4.96	2.98	**18.92**	34.85

After*								
V_{cmax}	13	15	22	12	5	6	**12**	6
J	38	38	44	30	12	20	**30**	12
TPU	2.1	2.5	2.3	2.3			**2.29**	0.17
R_d*								
g_m*								
α_G								
α_S								
SSR	12.12	7.46	8.92				**9.50**	2.38

Some parameters could not be determined because of very low stomatal conductance.

Colour	Meaning
Blue	Estimates are minimum of
Yellow	Minor TPU seen
Red	Significant TPU seen
